# Interannual and Cultivar-Related Variation in Sunflower Productivity and Seed Quality Under Long-Term Rainfed No-Till Management in the Semi-Arid Dobrogea Region of Romania

**DOI:** 10.3390/plants15203089

**Published:** 2026-10-09

**Authors:** Traian Ciprian Stroe, Anca Becze, Liliana Miron, Liliana Panaitescu

**Affiliations:** 1 Department of Agriculture, Doctoral School of Engineering and Management of Plant and Animal Resources (IM-RVA), University of Agronomic Sciences and Veterinary Medicine of Bucharest, 59 Mărăști Boulevard, 011464 Bucharest, Romania; lilipanaitescu@gmail.com; 2 Department of Natural Sciences, Faculty of Natural and Agricultural Sciences, Ovidius University of Constanța, 1 University Alley, Campus Building B, 900470 Constanța, Romania; miron_stroe_liliana@yahoo.com; 3 National Institute for Research and Development of Optoelectronics INOE 2000, Research Institute for Analytical Instrumentation, 67 Donath Str., 400293 Cluj-Napoca, Romania; anca.naghiu@icia.ro

**Keywords:** *Helianthus annuus* L., climatic variability, precipitation, drought, no-till, seed yield, seed quality, semi-arid environment

## Abstract

Seasonal climatic variability represents a major constraint on sunflower (*Helianthus annuus* L.) production in semi-arid environments, yet interannual and cultivar-related variation under long-term no-till management remains insufficiently characterized. This study evaluated interannual and cultivar-related variation in sunflower productivity and seed quality during three consecutive growing seasons (2023–2025) under rainfed long-term no-till conditions in the Dobrogea Region of Romania. Three commercial hybrids (Averon SU, Demetra, and Helesun) were evaluated in a randomized complete block design with three replications, while agronomic management remained unchanged throughout the experimental period. Agronomic traits, yield components, and seed quality parameters were analyzed using analysis of variance, paired *t*-tests, Pearson’s correlation analysis, and partial redundancy analysis (RDA). Pronounced interannual climatic variability was accompanied by substantial differences in sunflower performance. Mean seed yield reached 2169.06 kg ha^−1^ in 2023, declined to 848.08 kg ha^−1^ in the drought-affected 2024 season, which had the lowest March–August precipitation total, and partially recovered to 1178.68 kg ha^−1^ in 2025. Similar seasonal responses were observed for plant density at harvest, seed weight per capitulum, thousand grain weight, test weight, and number of seeds per capitulum, whereas oil content remained comparatively stable (45.19–46.88%). Analysis of variance showed significant interannual variation across all evaluated traits, while significant cultivar effects were detected for plant density, number of seeds per capitulum, seed weight per capitulum, thousand grain weight, and seed yield. Partial RDA further detected a significant joint cultivar effect across the eight standardized response traits after conditioning on study year and block (F = 11.49, R^2^ = 0.53, exact *p* = 0.027). Under the specific environmental conditions observed during the three study years, interannual variation in sunflower performance was substantial; however, annual climatic conditions were not independently replicated and therefore remain confounded with year. Consequently, the observed relationships describe associations within the 2023–2025 site conditions rather than establishing a general causal ranking of climatic and cultivar effects. The results highlight the importance of improving rainfall-use efficiency and integrating cultivar selection with climate-adaptive agronomic management to increase the resilience of sunflower production in water-limited environments.

## 1. Introduction

The global demand for vegetable oils has increased steadily over recent decades, reinforcing the importance of sunflower (*Helianthus annuus* L.) as one of the major oilseed crops worldwide. In addition to its substantial contribution to edible oil production, sunflower provides high-quality protein meal for livestock and valuable raw materials for the food, pharmaceutical, and bio-based industries. Sunflower seeds contain substantial amounts of unsaturated fatty acids, proteins, vitamins, and antioxidant compounds, which contribute to their nutritional and commercial value [1]. Continuous breeding efforts have further expanded the adaptability of sunflower through the development of hybrids with improved yield potential, enhanced oil quality, and greater resistance to biotic and abiotic stresses [2]. Advances in sunflower genetic improvement, from earlier applications of genetic engineering [3] to recent multi-trait hybrid breeding strategies [4], have progressively broadened the opportunities for improving crop performance and seed quality. Recent genotype evaluations further demonstrate the considerable genetic variability that remains available for yield and yield-related traits, emphasizing the importance of selecting plant material adapted to specific production environments [5]. Nevertheless, increasing climatic variability continues to challenge the capacity of modern hybrids to maintain stable productivity under water-limited field conditions.

Seasonal climatic conditions strongly regulate sunflower growth and productivity because plant responses to water availability and temperature vary throughout phenological development. Variations in rainfall amount and distribution, together with changes in thermal regimes, can affect crop establishment, vegetative growth, flowering, seed filling, and final oil accumulation. Ref. [6] demonstrated that fluctuations in precipitation and temperature account for a considerable proportion of the interannual variability in sunflower production across Central and Eastern Europe. Water deficit during flowering and seed filling can reduce photosynthetic activity, restrict assimilate translocation, and shorten the effective seed-filling period, ultimately decreasing seed number, individual seed weight, and final yield [7]. Substantial reductions in yield components and harvest index under drought conditions have also been reported by [8], while [9] documented pronounced morphological responses to limited soil water availability. Climatic stress may additionally affect seed composition, as the combination of drought and elevated temperatures can alter fatty acid composition and constrain oil accumulation [10]. Comparable responses have recently been reported under Romanian environmental conditions, where prolonged drought and recurrent heat waves have substantially reduced sunflower productivity and increased production uncertainty [11,12]. More recent evidence from Romania covering the 2020–2025 period has further demonstrated pronounced temporal and spatial fluctuations in sunflower seed yield and thousand-seed weight, with particularly unfavorable performance during the 2024 and 2025 growing seasons [13]. Understanding the relationships between these environmental constraints and sunflower performance is therefore essential for improving production stability under increasingly variable climatic conditions.

Conservation agriculture represents an important management approach for crop production in water-limited environments because reduced soil disturbance and surface residue retention can improve soil functioning and water conservation. No-till systems minimize mechanical soil disturbance, maintain crop residues at the soil surface, and may reduce evaporation while improving water infiltration [14]. Crop sequence under no-till management can influence sunflower establishment and subsequent crop performance [15], while reduced tillage has been associated with improvements in emergence, yield, and production efficiency [16]. Ref. [17] similarly highlighted the potential of conservation tillage to maintain sunflower productivity while improving the sustainability of cropping systems. Under Romanian conditions, recent studies have shown that no-till management can improve soil physical and hydro-physical characteristics and promote greater structural stability, attributes that may be particularly important when rainfall is limited or irregularly distributed [18,19]. The agronomic suitability of conservation agriculture under the pedoclimatic conditions of Dobrogea has also been reported for rapeseed [20], while reduced soil disturbance has been associated with changes in biological activity and nutrient cycling that may contribute to climate-adaptive agricultural management [21,22]. Evidence from long-term field experiments conducted within the same agricultural region further supports the suitability of conservation systems under increasingly variable climatic conditions [23]. Moreover, within diversified crop rotations, the inclusion of legumes before sunflower may contribute to soil fertility and more efficient resource utilization [24]. In the present study, however, no-till was maintained as a constant long-term management system rather than evaluated as an experimental treatment, providing a consistent agronomic background against which seasonal and cultivar-related variation could be assessed.

The Dobrogea Region of south-eastern Romania provides a particularly relevant environment for investigating these relationships because extensive sunflower cultivation coincides with pronounced water limitation and recurrent climatic stress. Dobrogea has been identified as one of Romania’s driest agricultural regions, characterized by a persistent negative climatic water balance and recurrent drought episodes [25]. Sunflower is among the dominant field crops cultivated in the region because of its comparatively good adaptation to dry environments and considerable economic importance [26]. Nevertheless, climatic instability has become increasingly evident, resulting in pronounced fluctuations in crop productivity and greater production risk [27]. Future climatic scenarios are expected to intensify these constraints [28], while substantial agricultural production losses associated with the combined effects of drought, heat stress, and extreme weather events have already been documented in the region [29]. Sunflower has consequently been recognized among the Romanian field crops particularly affected by recent climatic anomalies [11]. Recent multi-year assessments further indicate substantial year-to-year variation in sunflower performance across Romania, including Constanța County, reinforcing the importance of local environmental conditions in determining realized crop productivity [13]. These characteristics make the semi-arid Dobrogea Region a suitable field environment for examining how sunflower productivity and seed quality respond to contrasting growing seasons under a consistent conservation-oriented management system.

Multi-year field evaluations are particularly important for distinguishing consistent cultivar performance from responses that vary among growing seasons. In rainfed environments, the expression of yield and yield-related traits reflects the combined influence of genotype and the environmental conditions encountered during crop development. Consequently, cultivar rankings and the magnitude of differences among cultivars may change across years, resulting in genotype × environment interactions that are relevant for cultivar adaptation and production stability. Evaluating cultivars across contrasting growing seasons can therefore provide information not only on their average performance but also on the consistency of their responses under variable environmental conditions. However, when climatic conditions are shared by all experimental plots within a given year, year-specific environmental effects cannot be considered independently replicated climatic treatments, and relationships between climatic variables and crop performance should be interpreted as associations within the observed growing seasons.

Despite the considerable body of literature addressing drought stress, tillage systems, and sunflower cultivar performance, these components have frequently been investigated separately, limiting our understanding of their combined relationships with crop performance under practical farming conditions. Genotype evaluation remains fundamental for identifying productive and locally adapted sunflower material, and substantial differences among genotypes have been demonstrated under contrasting production environments [5]. Similarly, recent Romanian research continues to emphasize the need to develop and evaluate sunflower hybrids adapted to current environmental conditions [30]. However, genotype performance cannot be considered independently of environmental variability, particularly in water-limited production systems where year-to-year climatic fluctuations may substantially modify crop performance and cultivar responses. In particular, field-based evidence jointly examining interannual variation and cultivar responses under established rainfed no-till management remains limited for the semi-arid environments of South-Eastern Europe. Romanian studies have primarily addressed hybrid performance, crop management, or the effects of individual climatic events [31,32], whereas information jointly examining interannual environmental variation, cultivar performance, and seed quality under the specific pedoclimatic conditions of Dobrogea remains scarce [20]. Addressing this knowledge gap is important for understanding cultivar performance across contrasting growing seasons when sunflower crops are exposed to increasingly variable water and thermal regimes. Therefore, the present study aimed to evaluate interannual and cultivar-related variation in sunflower productivity, yield components, and seed quality under long-term rainfed no-till management in the semi-arid Dobrogea Region of Romania and to examine the associations between crop performance and the climatic conditions observed during the three study years. We hypothesized that sunflower performance would vary among growing seasons and cultivars and that the observed interannual variation would be associated with differences in seasonal water availability under otherwise consistent agronomic management.

## 2. Results

### 2.1. Climatic Conditions During the Experimental Period

**Figure 1:**
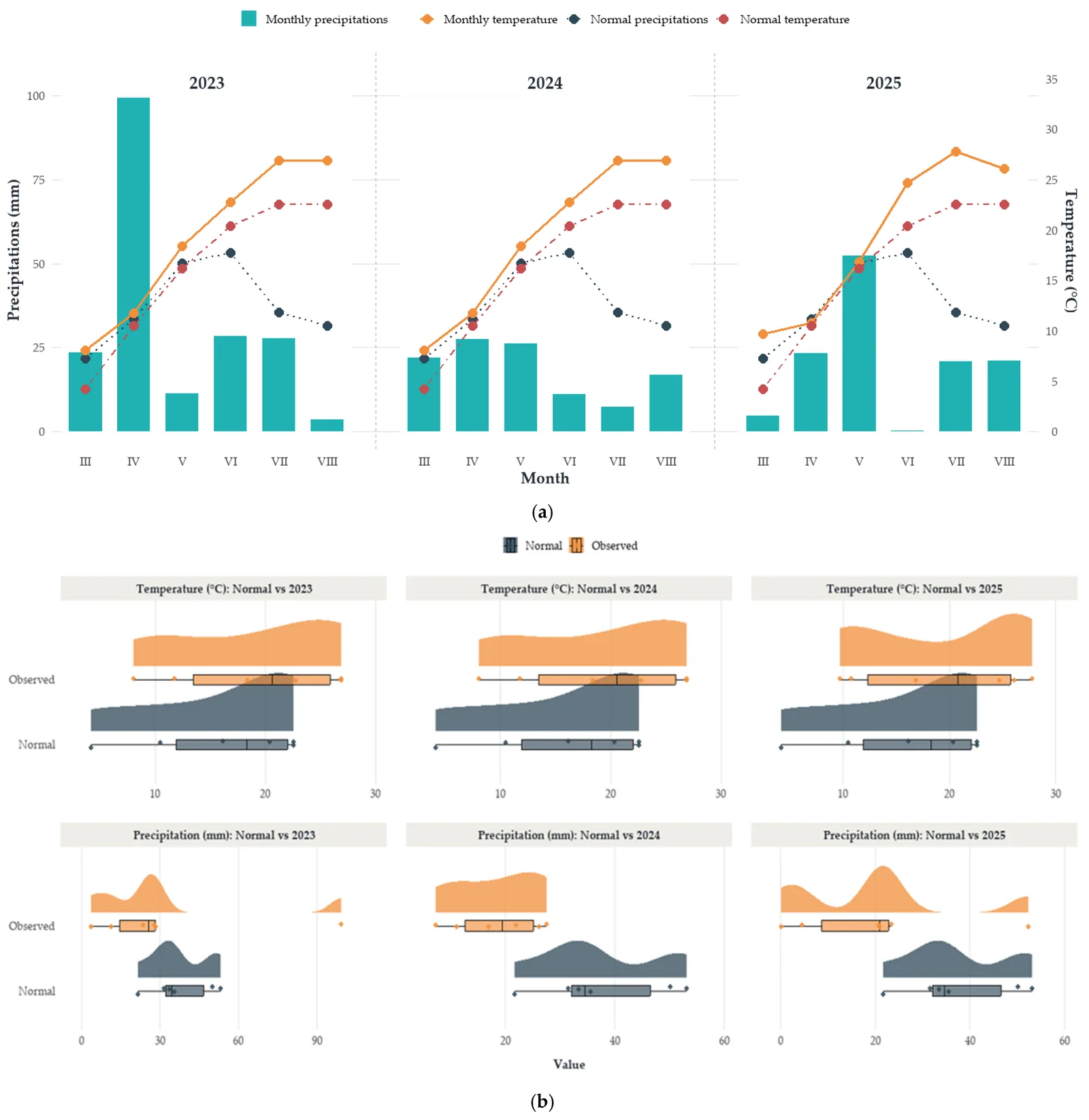
Interannual climatic variability during the 2023–2025 sunflower growing seasons in relation to long-term reference conditions. (**a**) Monthly precipitation and mean air temperature during the sunflower growing seasons (March–August) of 2023, 2024, and 2025 compared with the corresponding long-term climatic reference values. (**b**) Distribution of monthly precipitation and mean air temperature during the 2023–2025 sunflower growing seasons and the corresponding long-term reference period.

The three experimental growing seasons showed marked interannual variability in both precipitation and temperature during the March–August period (Figure 1). The highest cumulative precipitation was recorded in 2023, mainly due to the high rainfall registered in April (99.5 mm), followed by lower precipitation amounts during the summer months. In 2024, precipitation was lower and remained below the long-term reference values during most of the growing season. In 2025, rainfall was concentrated mainly in May (52.4 mm), while June was characterized by almost no precipitation (0.2 mm). Total precipitation during the March–August period reached 194.3 mm in 2023, compared with 111.4 mm in 2024 and 122.4 mm in 2025. Mean air temperature increased progressively from spring to summer in all three experimental years, with the highest monthly values recorded during July and August. The temperature patterns observed in 2023 and 2024 were relatively similar, while 2025 was characterized by higher summer temperatures, with mean values exceeding 24 °C in July and 27 °C in August.

Comparison with the long-term climatic reference values showed different patterns for precipitation and temperature. Precipitation varied considerably among months and experimental years, with the lowest March–August cumulative precipitation recorded in 2024 and with an uneven distribution also observed in 2025. In contrast, mean air temperatures during the experimental period were generally higher than the corresponding long-term reference values, particularly during the summer months (Figure 1a). Thus, the three study years represented contrasting combinations of precipitation amount, rainfall distribution, and thermal conditions during the sunflower growing period.

Mean monthly precipitation during March–August 2024 was significantly lower than the corresponding long-term reference values (mean paired difference = 19.07 mm, t = 3.01, *p* = 0.030). With a total precipitation of 111.4 mm from March to August, 2024 recorded the lowest cumulative precipitation of the three experimental seasons. In 2023 and 2025, precipitation differences from the long-term reference were not statistically significant (*p* = 0.740 and *p* = 0.070, respectively), although rainfall amount and monthly distribution differed between the two seasons.

Mean monthly temperature during March–August differed significantly from the corresponding long-term reference values in all three experimental years. In both 2023 and 2024, the mean difference was −3.07 °C (t = −5.90, *p* = 0.002), while in 2025 the mean difference reached −3.25 °C (t = −3.54, *p* = 0.017). These results indicate that above-reference temperatures were a common feature of all three growing seasons, despite the marked differences in precipitation among years.

The distribution of monthly precipitation and mean air temperature during the experimental period is further illustrated by the raincloud plots presented in Figure 1b. The plots show the distribution and variability of the climatic observations recorded during the three growing seasons and provide a complementary view of the interannual climatic differences observed during the experimental period.

Mean air temperature increased from spring to summer, with the highest monthly values recorded in July and August. Temperature patterns were similar in 2023 and 2024, while 2025 was characterized by higher summer temperatures, exceeding 24 °C in July and 27 °C in August. Compared with the long-term climatic averages, positive temperature anomalies were recorded during all three growing seasons, while precipitation showed greater differences among years.

Paired-sample *t*-tests were used to compare the monthly climatic values recorded during each growing season with the corresponding long-term reference values (Table 1). Mean air temperature differed significantly from the long-term reference in all three experimental years, with t = −5.90 and *p* = 0.002 in both 2023 and 2024, and t = −3.54 and *p* = 0.017 in 2025. These results indicate that higher-than-reference temperatures were a common characteristic of the three experimental seasons.

Precipitation showed a different pattern. A significant difference from the long-term reference values was detected only in 2024 (t = 3.01, *p* = 0.030), when the mean monthly difference reached 19.07 mm. No significant differences were detected for 2023 (t = 0.34, *p* = 0.740) or 2025 (t = 2.25, *p* = 0.070), despite the differences observed in total precipitation and its monthly distribution. Among the three experimental years, 2024 recorded the lowest March–August precipitation total (111.4 mm), compared with 194.3 mm in 2023 and 122.4 mm in 2025.

### 2.2. Seasonal and Cultivar-Related Variation in Agronomic Performance

**Figure 2:**
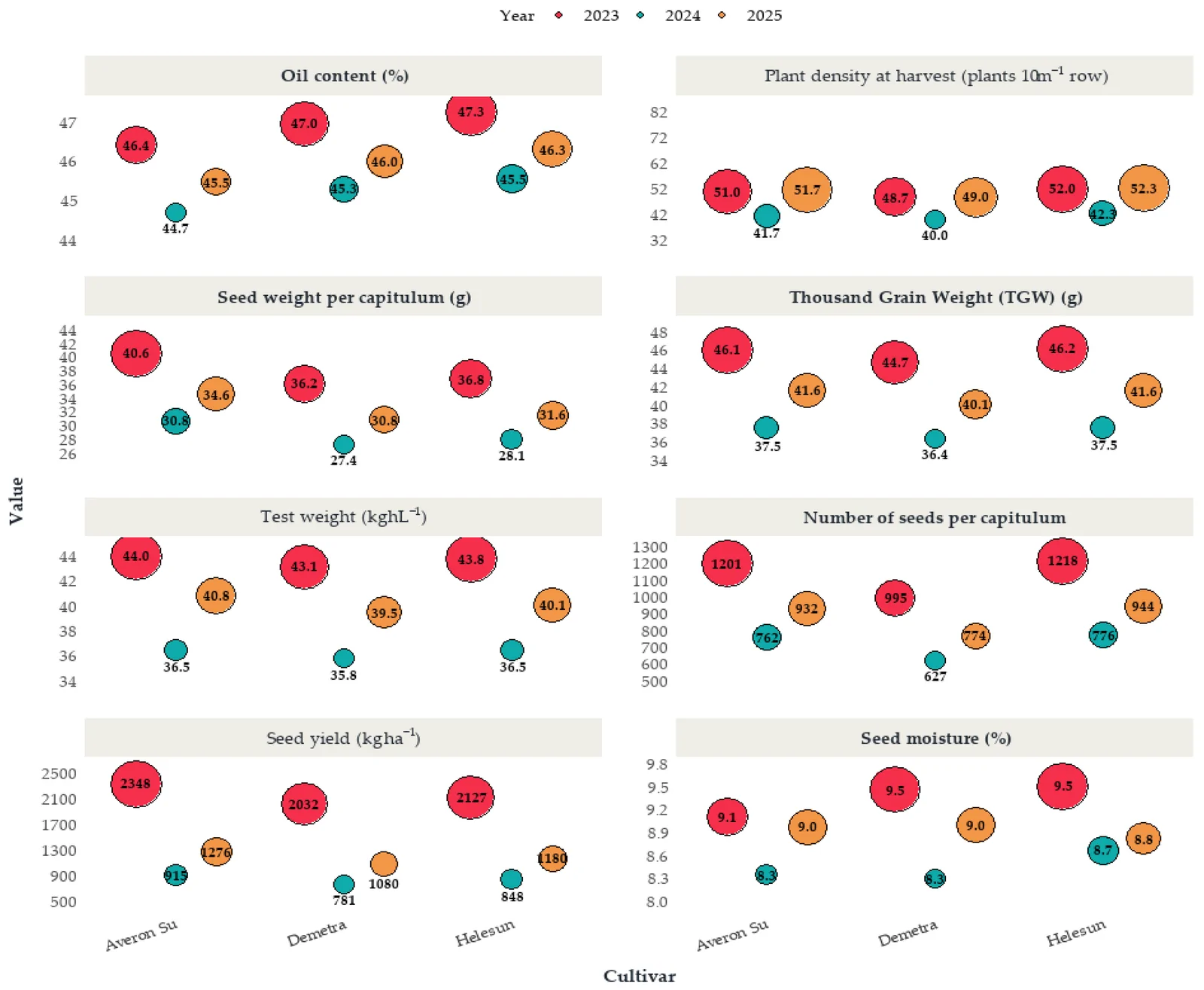
Parallel coordinate plot of agronomic, yield, and seed quality traits according to the three study years and sunflower cultivar.

The parallel coordinate plot showed a clear separation among the three growing seasons, while the profiles of the three sunflower cultivars showed a greater degree of overlap across the evaluated agronomic and seed quality traits (Figure 2). The 2023 growing season was associated with the highest values for most productivity-related parameters, while 2024 showed the lowest values. The response recorded in 2025 was generally intermediate between those observed in 2023 and 2024. The graphical pattern therefore illustrates pronounced interannual separation together with greater overlap among cultivar profiles, although trait-specific cultivar differences are evaluated statistically in the subsequent analyses.

Across the parallel coordinate plot, Averon SU, Demetra, and Helesun showed considerable overlap, whereas the profiles corresponding to the three study years were more clearly separated. The three cultivars followed broadly similar response patterns across several measured traits, although cultivar-specific differences were apparent for some productivity-related parameters. In contrast, the profiles corresponding to the three experimental years showed a distinct seasonal pattern, especially for seed yield, seed weight per capitulum, thousand grain weight, test weight, number of seeds per capitulum, and plant density at harvest. Seed quality traits, particularly oil content, showed smaller variation than the productivity-related parameters.

The graphical distribution of the measured traits therefore illustrates substantial interannual variation within the environmental conditions represented by the 2023–2025 experimental period, together with cultivar-related variation that differed among individual traits. The clearest interannual contrast was observed between 2023 and 2024, while the 2025 growing season generally showed an intermediate response.

### 2.3. Analysis of Variance of Agronomic and Seed Quality Traits

Mean agronomic and seed quality traits varied across the three study years and among cultivars, although the magnitude of these differences depended on the trait considered (Table 2). Across study years, the largest numerical contrasts were generally observed between 2023 and 2024, whereas 2025 showed intermediate values for several productivity and seed quality traits. Cultivar means also differed for several traits, particularly plant density at harvest, number of seeds per capitulum, seed weight per capitulum, thousand-grain weight (TGW), and seed yield.

The statistical significance of these differences was evaluated using the repeated-measures analysis summarized in Table 3. Significant differences among the three study years were detected for all eight agronomic and seed quality traits. Significant cultivar effects were detected for plant density at harvest (F_2,4_ = 11.33, *p* = 0.0225), number of seeds per capitulum (F_2,4_ = 38.44, *p* = 0.00245), seed weight per capitulum (F_2,4_ = 44.54, *p* = 0.00185), thousand-grain weight (TGW; F_2,4_ = 36.20, *p* = 0.00274), and seed yield (F_2,4_ = 71.51, *p* = 0.000740). In contrast, cultivar effects were not significant for test weight (*p* = 0.0816), seed moisture (*p* = 0.1187), or oil content (*p* = 0.0587). Significant cultivar × year interactions were detected for number of seeds per capitulum (Greenhouse–Geisser-adjusted F_2.18,6.53_ = 11.17, *p* = 0.00729), seed weight per capitulum on the original scale (F_2.47,7.40_ = 6.98, *p* = 0.0170), and seed yield (F_3.53,10.60_ = 56.90, *p* < 0.001). However, the cultivar × year interaction for seed weight per capitulum was not retained after log transformation (*p* = 0.619), indicating that this interaction was sensitive to response scale.

Oil content differed among the three study years (Greenhouse–Geisser-adjusted F_1.08,6.48_ = 4915.20, *p* < 0.001), with the highest mean recorded in 2023 (46.88%), followed by 2025 (45.92%) and 2024 (45.19%) (Table 2). Tukey-adjusted comparisons separated the three study-year means. In contrast, the cultivar effect was not statistically significant (*p* = 0.0587), although mean oil content ranged from 45.53% in Averon SU to 46.37% in Helesun. No cultivar × year interaction was detected (*p* = 0.6421).

Plant density at harvest differed significantly among study years (Greenhouse–Geisser-adjusted F_1.06,6.34_ = 185.56, *p* < 0.001). The highest mean density was recorded in 2025 (51.00 plants 10 m^−1^ row), followed closely by 2023 (50.56 plants 10 m^−1^ row), whereas 2024 had the lowest mean density (41.33 plants 10 m^−1^ row). A significant cultivar effect was also detected (F_2,4_ = 11.33, *p* = 0.0225), with Helesun recording the highest mean density (48.89 plants 10 m^−1^ row), Demetra the lowest (45.89 plants 10 m^−1^ row), and Averon SU an intermediate value (48.11 plants 10 m^−1^ row) (Table 2). The cultivar × year interaction was not significant (*p* = 0.8205).

Seed weight per capitulum differed significantly among study years (Greenhouse–Geisser-adjusted F_1.23,7.40_ = 4382.34, *p* < 0.001), with the highest mean recorded in 2023 (37.88 g), followed by 2025 (32.36 g) and 2024 (28.77 g). A significant cultivar effect was also detected (F_2,4_ = 44.54, *p* = 0.00185). Averon SU had the highest overall mean (35.34 g), whereas Helesun and Demetra averaged 32.18 and 31.49 g, respectively (Table 2). The cultivar × year interaction was significant on the original scale (F_2.47,7.40_ = 6.98, *p* = 0.0170); however, it was not retained after log transformation (*p* = 0.619). This interaction was therefore considered scale-sensitive and was interpreted cautiously.

Thousand-grain weight showed significant differences among study years (Greenhouse–Geisser-adjusted F_1.48,8.87_ = 8500.73, *p* < 0.001). The highest TGW was recorded in 2023 (45.67 g), followed by 2025 (41.12 g), while the lowest value occurred in 2024 (37.14 g), with all three study-year means separated by the Tukey-adjusted comparisons. Cultivar also had a significant effect (F_2,4_ = 36.20, *p* = 0.00274). Helesun (41.79 g) and Averon SU (41.73 g) had similar mean TGW values, both exceeding that of Demetra (40.41 g) (Table 2). The cultivar × year interaction was not significant (*p* = 0.1162).

Test weight differed significantly among study years (Greenhouse–Geisser-adjusted F_1.38,8.29_ = 392.17, *p* < 0.001), decreasing from 43.64 kg hL^−1^ in 2023 to 36.28 kg hL^−1^ in 2024 and reaching an intermediate value of 40.13 kg hL^−1^ in 2025. In contrast, the cultivar effect was not statistically significant (*p* = 0.0816), with mean values ranging from 39.48 kg hL^−1^ in Demetra to 40.44 kg hL^−1^ in Averon SU. No cultivar × year interaction was detected (*p* = 0.7463).

The number of seeds per capitulum differed significantly among study years (Greenhouse–Geisser-adjusted F_1.09,6.53_ = 3217.18, *p* < 0.001), with the highest mean recorded in 2023 (1138.00), followed by 2025 (883.44), and the lowest in 2024 (721.89). A significant cultivar effect was detected (F_2,4_ = 38.44, *p* = 0.00245). Helesun and Averon SU recorded means of 979.67 and 965.00 seeds per capitulum, respectively, whereas Demetra had a lower mean of 798.67 seeds per capitulum (Table 2). A significant cultivar × year interaction was also detected (Greenhouse–Geisser-adjusted F_2.18,6.53_ = 11.17, *p* = 0.00729), indicating that the magnitude of cultivar differences in seed number varied among the three study years.

Seed yield differed markedly among study years (Greenhouse–Geisser-adjusted F_1.77,10.60_ = 31,082.38, *p* < 0.001), with a mean of 2169.06 kg ha^−1^ in 2023, compared with 848.08 kg ha^−1^ in 2024 and 1178.68 kg ha^−1^ in 2025. Cultivar also had a significant effect on seed yield (F_2,4_ = 71.51, *p* = 0.000740). Across the three study years, Averon SU had the highest mean yield (1513.06 kg ha^−1^), followed by Helesun (1385.22 kg ha^−1^) and Demetra (1297.55 kg ha^−1^) (Table 2). The cultivar × year interaction was significant (Greenhouse–Geisser-adjusted F_3.53,10.60_ = 56.90, *p* < 0.001), showing that the magnitude of the yield differences among cultivars was not constant across study years. Accordingly, the cultivar-specific yield responses within individual study years are considered separately in the subsequent interaction analysis.

Seed moisture differed significantly among study years (Greenhouse–Geisser-adjusted F_1.76,10.55_ = 10.52, *p* = 0.00373). The highest mean moisture was recorded in 2023 (9.36%), the lowest in 2024 (8.44%), and an intermediate value in 2025 (8.94%). The cultivar effect was not statistically significant (*p* = 0.1187), with overall means ranging only from 8.81% in Averon SU to 9.00% in Helesun. The cultivar × year interaction was also not significant (*p* = 0.6609).

The repeated-measures analysis demonstrated substantial interannual variation in all evaluated traits under the environmental conditions represented by the 2023–2025 study period, while cultivar-related differences were trait-dependent. Significant cultivar effects were identified for five of the eight traits, and significant cultivar × year interactions were detected for seed number and seed yield, with a scale-sensitive interaction additionally observed for seed weight per capitulum. These results characterize variation among the three observed study years and should not be interpreted as providing an independently replicated estimate of climatic effects.

### 2.4. Relative Response of Agronomic and Seed Quality Traits

**Figure 3:**
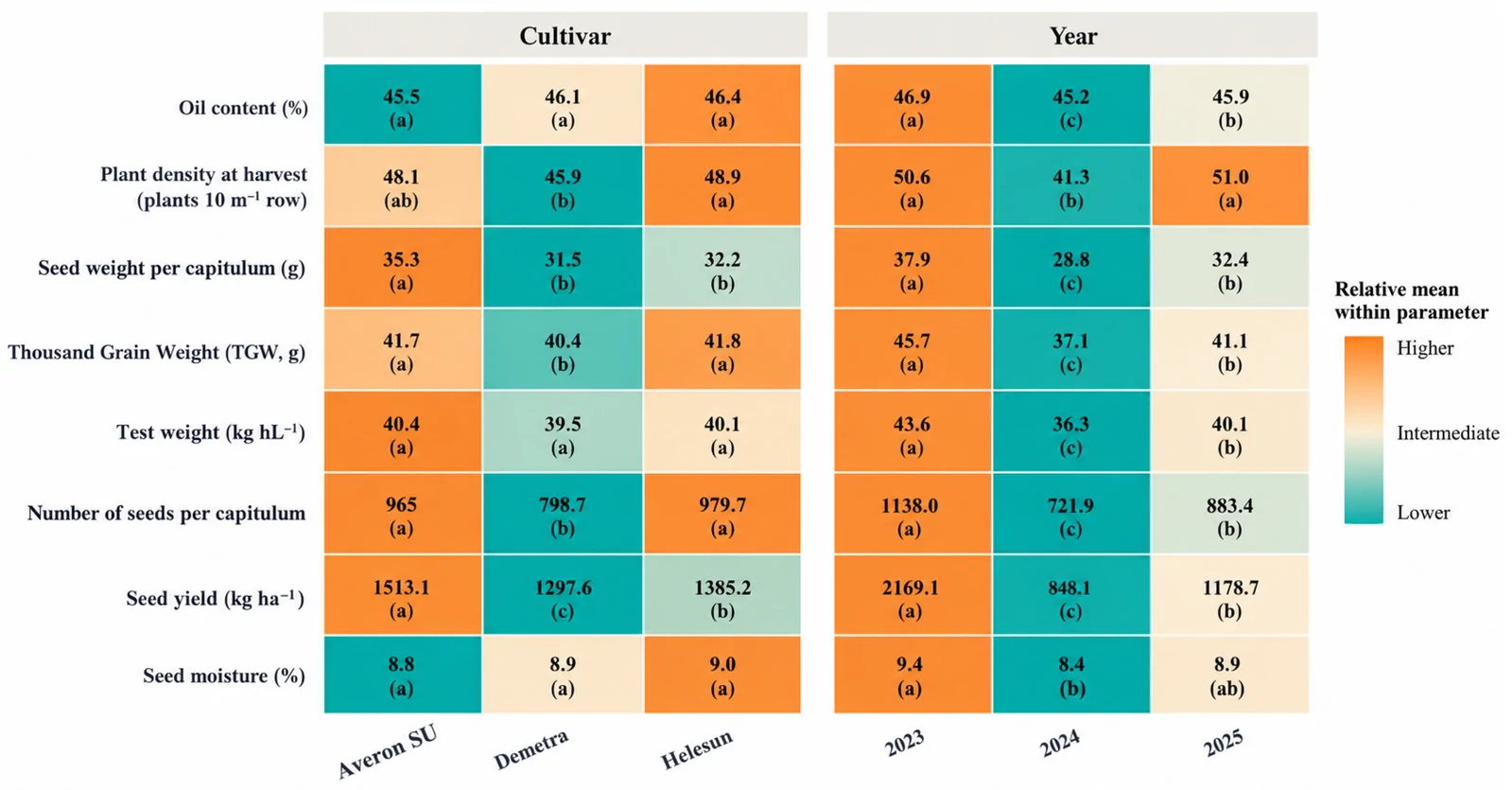
Heatmap of mean agronomic and seed quality traits according to sunflower cultivar and study year during the 2023–2025 experimental period. Cell values represent the mean of each parameter, while the color scale indicates the relative magnitude of the mean within each trait, from lower to higher values. Within each factor (cultivar and study year), different lowercase letters indicate significant differences according to Tukey-adjusted pairwise comparisons at *p* < 0.05.

To provide an integrated comparison of the measured variables, mean agronomic and seed quality traits were visualized using a heatmap (Figure 3). The graphical representation summarizes the interannual and cultivar-related variation observed across the 2023–2025 experimental period. The three study years showed distinct profiles for most traits, while the magnitude of cultivar-related differences varied among individual parameters.

Thousand-grain weight (TGW) showed clear interannual variation, with the highest mean recorded in 2023 (45.67 g), followed by 2025 (41.12 g), and the lowest value in 2024 (37.14 g). Cultivar-related differences were also evident, with Helesun (41.79 g) and Averon SU (41.73 g) showing similar mean values, both higher than Demetra (40.41 g). Thus, the heatmap illustrates both the separation among study years and the cultivar-related differences detected for this trait.

Test weight showed a similar interannual pattern, reaching its highest mean in 2023 (43.64 kg hL^−1^), decreasing to 36.28 kg hL^−1^ in 2024, and reaching an intermediate value of 40.13 kg hL^−1^ in 2025. In contrast to TGW, differences among cultivars were not statistically significant, with mean values ranging from 39.48 kg hL^−1^ for Demetra to 40.44 kg hL^−1^ for Averon SU.

The number of seeds per capitulum also varied among study years, with the highest mean recorded in 2023 (1138.00), followed by 2025 (883.44), and the lowest value in 2024 (721.89). Cultivar-related differences were also detected, with Helesun (979.67) and Averon SU (965.00) showing higher overall means than Demetra (798.67). As indicated by the significant cultivar × year interaction reported in Table 3, however, the magnitude of these cultivar differences varied among study years.

Seed yield showed one of the clearest interannual contrasts. The highest mean yield was recorded in 2023 (2169.06 kg ha^−1^), whereas the mean decreased to 848.08 kg ha^−1^ in 2024, corresponding to an approximately 61% reduction relative to 2023. In 2025, mean seed yield increased to 1178.68 kg ha^−1^ but remained below the 2023 value. Across the three study years, Averon SU had the highest overall mean yield (1513.06 kg ha^−1^), followed by Helesun (1385.22 kg ha^−1^) and Demetra (1297.55 kg ha^−1^). However, because a significant cultivar × year interaction was detected for seed yield (Table 3), these overall cultivar means should be interpreted together with the cultivar-specific responses within individual study years.

The significant cultivar × year interaction detected for seed yield (Table 3) indicated that the magnitude of cultivar differences varied among study years; therefore, cultivar-specific yield responses within each study year were examined separately (Table 4).

The year-specific cultivar means showed a consistent numerical ranking across the three study years (Table 4). Averon SU recorded the highest mean seed yield in each year, reaching 2348.02 kg ha^−1^ in 2023, 915.15 kg ha^−1^ in 2024, and 1275.99 kg ha^−1^ in 2025. Helesun showed intermediate values of 2127.23, 848.01, and 1180.42 kg ha^−1^, respectively, whereas Demetra recorded the lowest numerical means, with 2031.94, 781.09, and 1079.63 kg ha^−1^. Although this numerical ranking was consistent, the magnitude of the differences among cultivars varied across study years, consistent with the significant cultivar × year interaction detected in the repeated-measures analysis.

The heatmap illustrates substantial interannual variation across the evaluated agronomic and seed quality traits, with 2023 generally characterized by higher productivity-related values, 2024 by lower values, and 2025 by intermediate or partially recovered values. Cultivar-related variation was trait-dependent, with significant differences detected for several productivity-related parameters. Moreover, the significant cultivar × year interactions observed for seed number and seed yield indicate that cultivar differences were not uniform across the three study years. These graphical patterns complement the repeated-measures analysis and the year-specific cultivar comparisons presented in Table 3 and Table 4, respectively, without implying independently replicated climatic effects.

### 2.5. Cultivar-Specific Relative Responses Across Study Years

**Figure 4:**
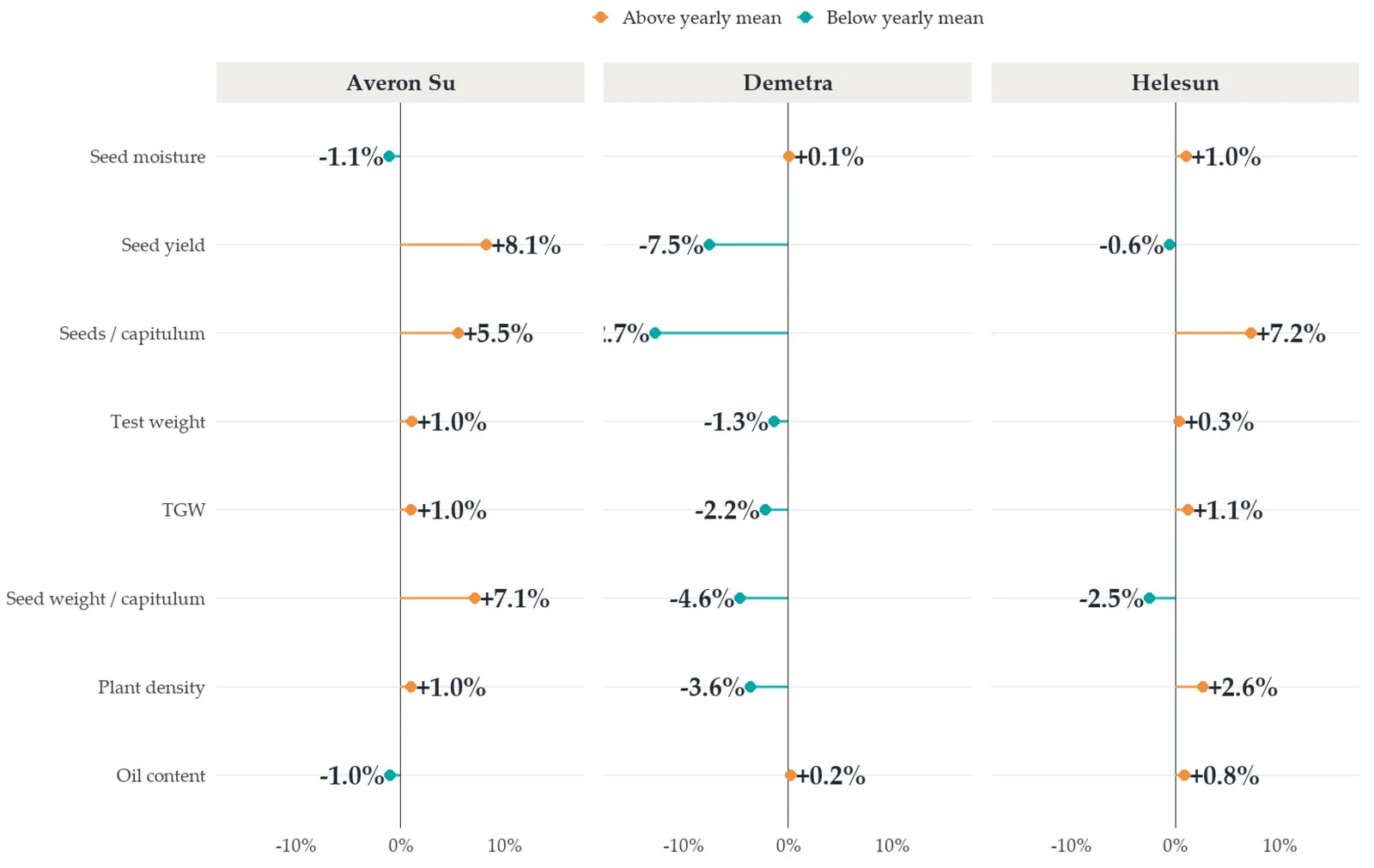
Mean cultivar deviations from the corresponding yearly means for eight sunflower agronomic and seed quality traits across the 2023–2025 study period. Positive values indicate cultivar means above the corresponding yearly mean, whereas negative values indicate means below it. Deviations were calculated separately for each trait and study year and subsequently averaged across the three study years. The relative deviations are descriptive and do not represent separate tests of statistical significance.

To visualize cultivar-specific patterns relative to the corresponding yearly means, cultivar means were expressed as percentage deviations from the corresponding yearly mean for each trait and subsequently averaged across the three study years (Figure 4). These relative deviations provide a descriptive representation of cultivar performance after expressing each cultivar relative to the common mean of the corresponding study year.

Averon SU showed positive relative deviations for several productivity-related traits. Positive deviations were observed for seed yield (+8.1%), seed weight per capitulum (+7.1%), number of seeds per capitulum (+5.5%), plant density (+1.0%), thousand grain weight (TGW; +1.0%), and test weight (+1.0%). Oil content (−1.0%) and seed moisture (−1.1%) were only slightly below the corresponding yearly means.

Demetra showed predominantly negative deviations for productivity-related traits. The largest negative deviations were recorded for the number of seeds per capitulum (−12.7%) and seed yield (−7.5%), followed by seed weight per capitulum (−4.6%), plant density (−3.6%), TGW (−2.2%), and test weight (−1.3%). In contrast, oil content (+0.2%) and seed moisture (+0.1%) remained close to the corresponding yearly means.

Helesun showed positive deviations for the number of seeds per capitulum (+7.2%), plant density (+2.6%), TGW (+1.1%), seed moisture (+1.0%), oil content (+0.8%), and test weight (+0.3%). Seed yield remained close to the yearly mean (−0.6%), while seed weight per capitulum showed a negative deviation (−2.5%).

The relative deviation profiles therefore illustrate distinct cultivar-specific patterns across the evaluated traits. Averon SU showed positive relative deviations for several productivity-related traits, Demetra showed predominantly negative deviations for most yield-related parameters, and Helesun remained generally close to corresponding yearly means while showing positive deviations for several individual traits. These profiles are descriptive summaries of relative cultivar performance and do not constitute separate statistical tests. Statistical inference regarding cultivar effects and cultivar × year interactions is provided by the repeated-measures analysis presented in Table 3.

### 2.6. Relationships Between Seed Yield and Agronomic and Seed Quality Traits

**Figure 5:**
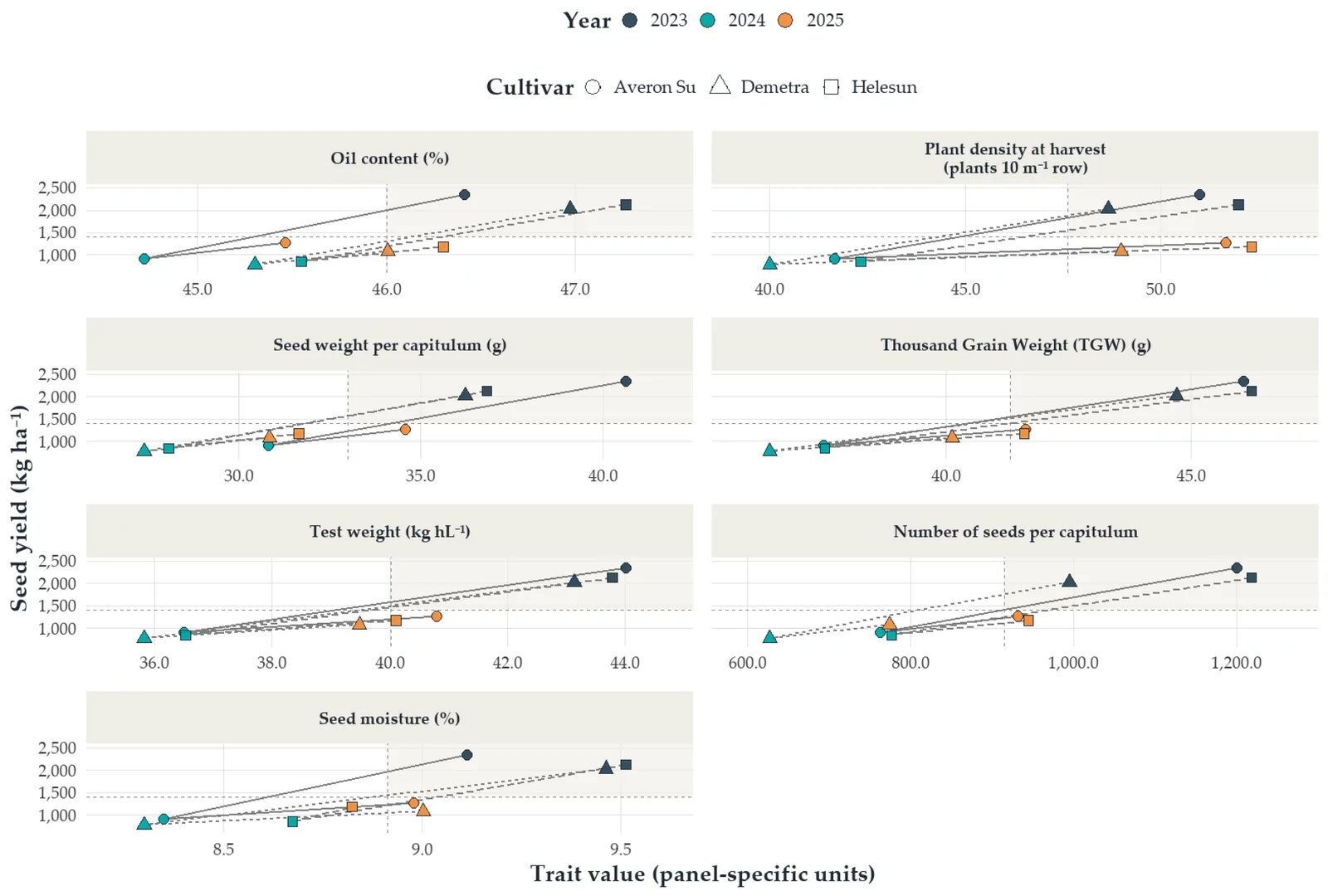
Relationships between seed yield and seven agronomic and seed quality traits across sunflower cultivars and study years (2023–2025). Colors represent study years, symbols identify cultivars, and connecting lines show the trajectory of each cultivar across the three study years. Dashed horizontal and vertical lines indicate the overall mean seed yield and the overall mean of the corresponding trait, respectively. The relationships are presented descriptively and do not represent independent tests of climatic effects.

To examine the graphical relationships between seed yield and the measured agronomic and seed quality traits, seed yield was plotted against each parameter across cultivars and study years (Figure 5). Distinct interannual patterns were apparent across most relationships, with the 2023 observations combining higher seed yields with higher values of seed weight per capitulum, thousand-grain weight (TGW), test weight, and number of seeds per capitulum.

In 2024, lower seed yield coincided with lower values of several productivity-related traits, whereas 2025 showed a partial recovery. Seed yield followed broadly similar interannual patterns to seed weight per capitulum, TGW, test weight, and number of seeds per capitulum. Plant density showed a different pattern, recovering in 2025 to a mean value comparable to that recorded in 2023, while seed yield remained below the 2023 level. Cultivar-specific trajectories were also apparent in several panels, consistent with the trait-dependent cultivar effects and cultivar × year interactions identified by the repeated-measures analysis. The graphical relationships shown in Figure 5 are descriptive and illustrate covariation among traits within the three observed study years rather than independent climatic effects.

### 2.7. Integrated Interannual Responses

**Figure 6:**
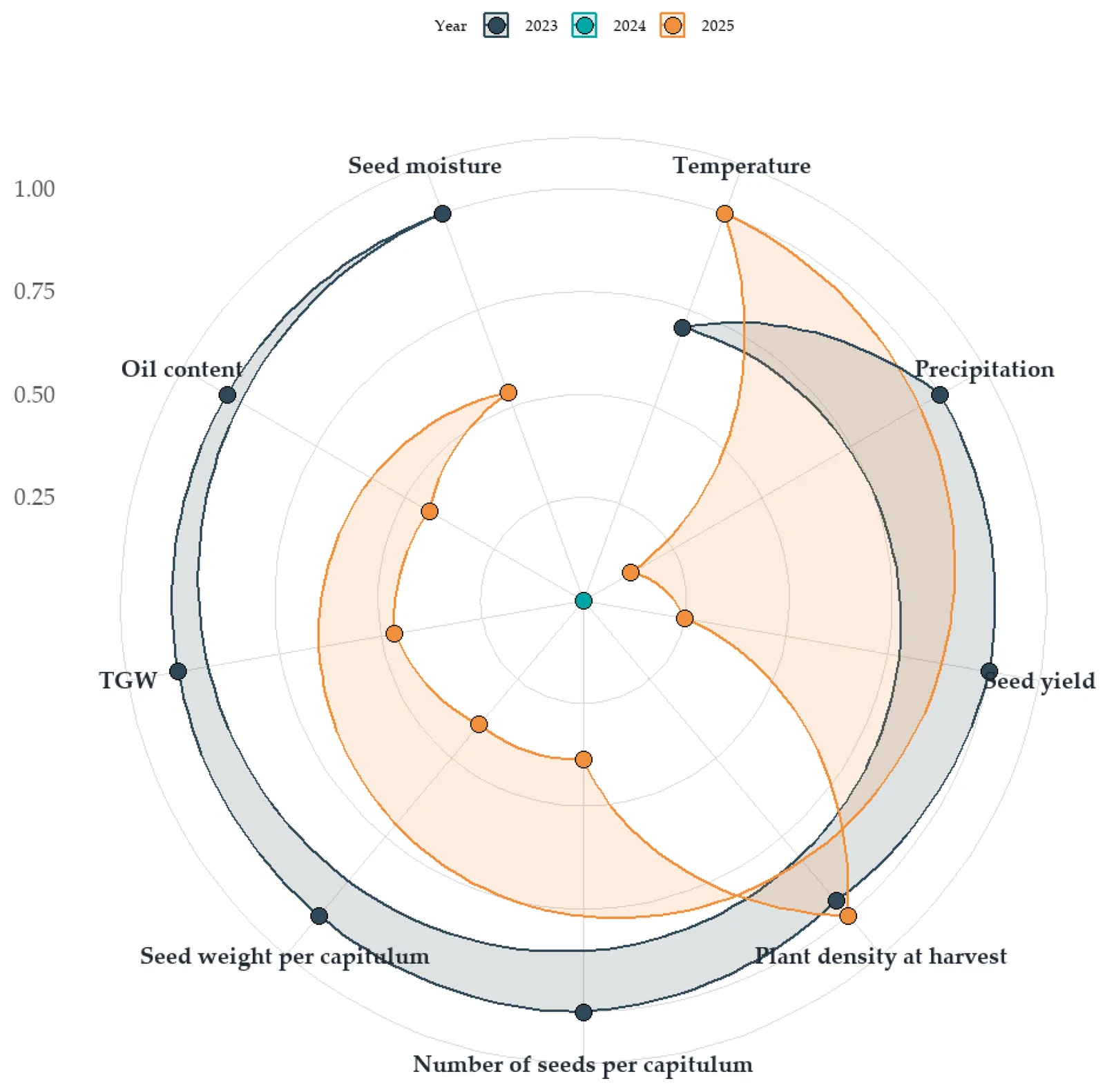
Radar plot showing the normalized climatic, agronomic, yield, and seed quality variables across the three study years (2023–2025). Values were normalized to allow descriptive comparison among variables expressed in different measurement units. Precipitation and mean air temperature represent year-level environmental conditions and are included for descriptive purposes only.

The radar plot provides an integrated descriptive comparison of the normalized climatic, agronomic, yield, and seed quality variables across the three study years (Figure 6). Distinct profiles were observed among the three study years, with 2023 showing higher normalized values for most productivity-related traits, 2024 showing lower values, and 2025 generally occupying an intermediate position. Climatic variables are included to characterize the environmental conditions of each study year and are not treated as independently replicated explanatory variables.

Among the three study years, 2023 showed the highest normalized values for most productivity-related traits and was also characterized by the highest precipitation total. Higher values were recorded for seed weight per capitulum, thousand-grain weight (TGW), test weight, number of seeds per capitulum, seed yield, oil content, and seed moisture, while plant density at harvest was also among the highest values observed during the experimental period. The combined pattern resulted in the broadest radar profile for most productivity-related variables in 2023.

In contrast, 2024 showed the lowest normalized values for most productivity-related traits and was characterized by the lowest precipitation among the three study years. Lower values were recorded for plant density, seed weight per capitulum, TGW, test weight, number of seeds per capitulum, and seed yield. Oil content and seed moisture were also lower than in 2023. Consequently, 2024 produced the smallest radar profile for most productivity-related variables.

The 2025 study year showed an intermediate profile for most evaluated variables. Precipitation remained below the level recorded in 2023, whereas mean air temperature reached the highest normalized value among the three study years. Seed weight per capitulum, TGW, test weight, number of seeds per capitulum, and seed yield were higher than in 2024 but remained below the corresponding values recorded in 2023. Oil content and seed moisture showed a similar intermediate pattern. Plant density differed from this general response, reaching a level comparable to that recorded in 2023.

The integrated profiles therefore illustrate marked interannual variation across the three study years, particularly between 2023 and 2024, while 2025 generally showed an intermediate response. Differences were especially evident for productivity-related traits, whereas some seed quality parameters, particularly oil content, showed comparatively smaller variation. Within the specific environmental conditions observed during 2023–2025, variation in sunflower performance coincided with interannual differences in precipitation and temperature. Because these climatic variables were measured at the study-year level and were common to all plots within each year, the radar plot provides a descriptive visualization of these concurrent patterns and does not estimate independent climatic effects.

### 2.8. Multivariate Cultivar Effects and Descriptive Climatic Relationships

The partial redundancy analysis (partial RDA) of the eight standardized agronomic and seed quality traits detected a significant multivariate cultivar effect after conditioning on study year and block (pseudo-F_2,20_ = 11.49, partial R^2^ = 0.535, exact *p* = 0.0278). The exact design-based test was based on 216 permissible whole-plot allocations, including the observed allocation. This result indicates a joint cultivar-related signal across the multivariate response matrix after accounting for variation associated with study year and block.

Because precipitation and mean air temperature had only three unique year-level values and were common to all plots within each year, these variables were not subjected to inferential testing in the partial RDA, and no independent climatic effects were estimated.

**Figure 7:**
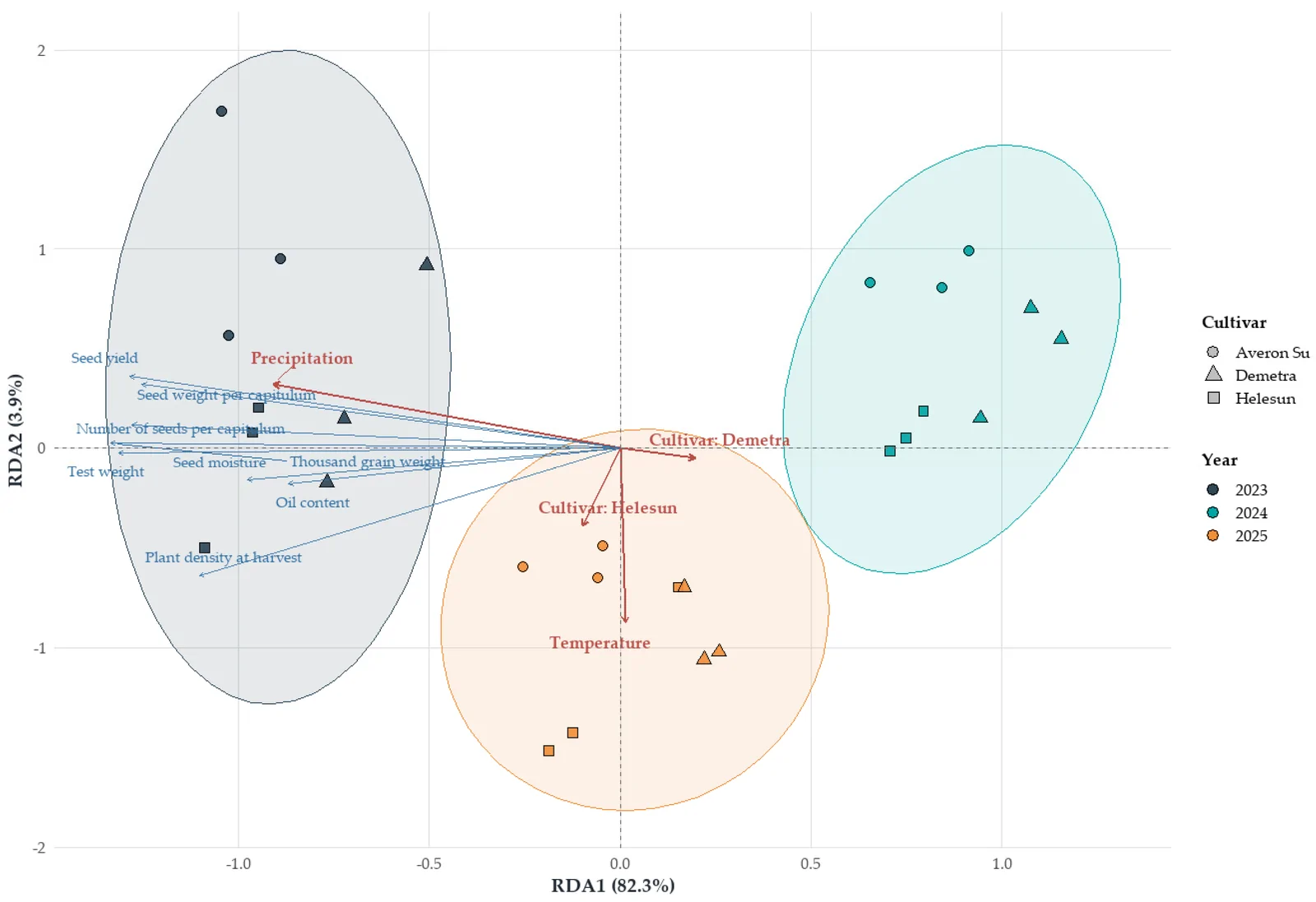
Descriptive redundancy analysis (RDA) biplot illustrating the multivariate correspondence among the environmental conditions of the three study years, sunflower cultivars, and standardized agronomic and seed quality traits across the 27 plot-year observations. Precipitation and mean air temperature had only three unique year-level values and were repeated across plots within each year for visualization purposes; therefore, the climate-related ordination was not used for permutation-based inference or estimation of independent climatic effects. Blue arrows represent agronomic and seed-quality response variables, whereas red arrows represent explanatory variables; ellipses group observations by year.

To complement the inferential cultivar analysis, the original climate-related RDA ordination was retained exclusively as a descriptive visualization of the multivariate correspondence among the environmental conditions of the three study years, cultivar identity, and sunflower performance traits (Figure 7). In this descriptive ordination, the model represented 88.29% of the total variation in the dataset (adjusted R^2^ = 86.16%); these values describe the ordination structure and are not interpreted as estimates of independently replicated climatic effects.

The first canonical axis (RDA1) explained 82.26% of the total variation and 93.17% of the constrained variation, while the second canonical axis (RDA2) accounted for an additional 3.89% of the total variation and 4.40% of the constrained variation. Together, the first two canonical axes therefore represented 86.15% of the total variation and 97.57% of the constrained variation. These percentages are reported solely to describe the distribution of variation within the descriptive ordination and were not subjected to inferential interpretation.

In the descriptive ordination, precipitation and temperature vectors were retained only to illustrate their correspondence with the multivariate trait structure across the three study years. Because both climatic variables varied exclusively at the year level, the lengths and orientations of these vectors were not interpreted as measures of independent climatic effects or as a quantitative comparison with cultivar effects.

The descriptive RDA biplot showed a clear separation among the three study years along the first canonical axis. Samples from 2023 were grouped on the negative side of RDA1 and were closely associated with the precipitation vector and most productivity-related variables, including seed yield, plant density at harvest, number of seeds per capitulum, seed weight per capitulum, thousand-grain weight (TGW), oil content, and seed moisture. The similar orientation of these vectors illustrates the positive correspondence between precipitation and these agronomic and seed quality traits within the three-year dataset. In contrast, observations from 2024 formed a distinct cluster separated from the 2023 samples and were positioned away from most of the productivity-related vectors, corresponding to the lower values recorded for the majority of these traits during that study year. Samples from 2025 occupied an intermediate position in the ordination space and were more closely aligned with the temperature vector, consistent with the higher temperatures recorded during this study year and the partial recovery observed for several agronomic traits compared with 2024.

The inferential partial RDA nevertheless detected a significant joint cultivar effect across the eight standardized response traits after conditioning on study year and block (pseudo-F_2,20_ = 11.49, partial R^2^ = 0.535, exact *p* = 0.0278). This multivariate cultivar signal should be interpreted independently of the descriptive climate-related ordination shown in Figure 7. It indicates that cultivar identity was associated with the joint multivariate response after accounting for year- and block-associated variation, but it does not provide an estimate of an independent precipitation or temperature effect.

## 3. Discussion

### 3.1. Interannual Climatic Variability and Sunflower Productivity

The present study showed pronounced interannual variation in sunflower productivity under rainfed no-till conditions in the semi-arid Dobrogea Region. Despite maintaining the same agronomic practices and crop management throughout the experimental period, substantial differences were recorded among the three study years, while cultivar-related responses were trait-dependent. The repeated-measures analysis detected significant cultivar effects for five of the eight evaluated traits, and the partial RDA further identified a significant joint multivariate cultivar effect after conditioning on study year and block (pseudo-F_2,20_ = 11.49, partial R^2^ = 0.535, exact *p* = 0.0278). Precipitation and mean air temperature, however, varied only at the study-year level and were common to all experimental plots within each year. Consequently, relationships between these climatic variables and sunflower performance should be interpreted as associations within the environmental conditions represented by the 2023–2025 study period rather than as independent estimates of climatic effects. Under these conditions, sunflower productivity showed substantial interannual variation associated with contrasting seasonal climatic conditions.

The contrast between 2023 and 2024 illustrates the magnitude of this interannual variation. The 2023 growing season received 194.3 mm of precipitation from March to August and produced the highest seed yield (2169.06 kg ha^−1^), together with higher values for most yield components. In 2024, precipitation during the same period decreased to 111.4 mm and seed yield declined to 848.08 kg ha^−1^, representing a reduction of approximately 61% compared with 2023. This decline was accompanied by reductions in plant density, number of seeds per capitulum, seed weight per capitulum, thousand grain weight, and test weight. The simultaneous decline in several yield components coincided with the lower seasonal water availability observed in 2024 and illustrates the coordinated interannual variation in multiple traits involved in yield formation.

These findings are consistent with previous studies showing the sensitivity of sunflower productivity to water availability, particularly during reproductive development. Ref. [7] demonstrated that drought during flowering and seed filling restricts cell-extension growth and seed filling, thereby reducing final seed development. Ref. [8] similarly reported substantial reductions in sunflower yield components and harvest index under drought stress, while [1,9] observed pronounced changes in morphological traits under limited water availability. At a broader geographical scale, ref. [6] identified agricultural drought as an important determinant of interannual variation in sunflower production, demonstrating the close relationship between seasonal water availability and crop performance.

The responses observed in the present experiment are also consistent with the climatic constraints previously reported for south-eastern Romania. Dobrogea has long been recognized as one of the driest agricultural regions of Romania, characterized by a persistent negative climatic water balance and recurrent drought episodes [25]. More recent studies indicate that climatic instability is becoming increasingly important for agricultural production in this region [27,28]. At the national level, ref. [11] reported substantial effects of recent climatic variability on sunflower and other major field crops, while [20] associated drought and extreme heat with considerable reductions in sunflower seed yield. The decline in sunflower productivity reported in Romania during the 2020–2025 period by [12] further emphasizes the relevance of these climatic constraints for current production systems.

An important pattern observed during the three study years was the close correspondence between precipitation availability and sunflower productivity. Mean temperatures differed significantly from the long-term reference values in all three experimental years, whereas the lowest sunflower productivity was recorded in 2024, which also had the lowest March–August precipitation total. In contrast, 2025 was characterized by higher temperatures but showed a partial recovery in several productivity traits compared with 2024. Within the environmental conditions represented by these three study years, this pattern is consistent with previously reported relationships between seasonal water availability and sunflower performance. However, because precipitation and temperature were not independently replicated and remained confounded with study year, their relative effects cannot be quantitatively separated from the present experimental design. Elevated temperatures may further increase crop water demand and intensify water deficits, particularly when rainfall is limited or unevenly distributed.

The RDA results were consistent with this observational pattern, as precipitation was closely aligned with seed yield and several major yield components, including seed weight per capitulum, number of seeds per capitulum, thousand grain weight, and plant density. However, because the annual climatic variables were shared by all plots within each study year, the ordination cannot be interpreted as providing an independent quantitative comparison between climatic and cultivar effects. Rather, it illustrates the multivariate associations among the climatic conditions and sunflower responses observed during the 2023–2025 experimental period. The univariate and multivariate analyses therefore provide complementary information on cultivar-related variation. The repeated-measures analysis evaluated each response trait separately and detected significant cultivar effects for plant density at harvest, number of seeds per capitulum, seed weight per capitulum, thousand-grain weight, and seed yield, whereas cultivar effects were not significant for test weight, seed moisture, or oil content. In contrast, the partial RDA evaluated the joint response across all eight standardized traits and detected a significant multivariate cultivar effect after conditioning on study year and block (pseudo-F_2,20_ = 11.49, partial R^2^ = 0.535, exact *p* = 0.0278). Thus, the significant partial RDA result reflects a joint cultivar-related signal across the multivariate trait structure and does not imply that every individual trait differed significantly among cultivars.

### 3.2. Differential Responses of Yield Components and Seed Quality

The present results showed more pronounced interannual variation in sunflower yield and its components than in seed oil content. Plant density at harvest, number of seeds per capitulum, seed weight per capitulum, thousand grain weight (TGW), test weight, and final seed yield exhibited pronounced year-to-year differences, whereas oil content varied within a considerably narrower range. Seed yield declined from 2169.06 kg ha^−1^ in 2023 to 848.08 kg ha^−1^ in 2024, while oil content decreased only from 46.88% to 45.19%. This contrasting response indicates that productivity was considerably more variable across the three study years than total seed oil concentration.

Yield formation in sunflower results from the successive development of several structural components throughout the growing season. Environmental stress occurring during different developmental stages may therefore affect more than one component of final productivity. In the present study, the lower yield recorded in 2024 was accompanied by reductions in plant density, number of seeds per capitulum, seed weight per capitulum, TGW, and test weight. The number of seeds per capitulum declined from 1138 in 2023 to 721.89 in 2024, while seed weight per capitulum decreased from 37.88 to 28.77 g and TGW from 45.67 to 37.14 g. These concurrent changes indicate that the reduction in final yield was accompanied by changes in several yield components rather than in a single trait. Ref. [33] similarly identified relationships between sunflower yield and head-related yield components, while [8] reported substantial reductions in yield components and harvest index under drought stress.

The response of seed quality was less pronounced. Oil content remained within a relatively narrow interval, ranging from 45.19% in 2024 to 46.88% in 2023, despite the much larger differences observed in seed yield and its components. This suggests that total oil concentration was comparatively stable across the three study years evaluated in the present study. Environmental stress can nevertheless influence lipid metabolism and seed composition. Ref. [10] demonstrated that drought stress modifies sunflower fatty acid composition and affects both grain yield and oil quality, indicating that relatively limited changes in total oil concentration do not necessarily imply an absence of changes in lipid composition.

The relative stability of oil content may also be considered in relation to the characteristics of modern sunflower germplasm. Current breeding programs increasingly combine productivity with oil quality, stress tolerance, and adaptation to contrasting environments [2]. Ref. [4] further emphasized multi-trait selection for yield and oil-related characteristics in modern sunflower breeding. In the present experiment, Averon SU, Demetra, and Helesun showed no significant differences in oil content, while the numerical range in oil content among the three study years was considerably narrower than the corresponding interannual variation observed for productivity-related traits. These findings suggest that seed oil concentration was relatively stable across the three cultivars and study years, although the three-year duration of the experiment does not allow this response to be generalized beyond the environmental conditions evaluated here.

The distinction between productivity and seed quality is relevant from both agronomic and technological perspectives. Sunflower is valued not only for seed production but also for its high-quality vegetable oil and nutritional composition [1]. Sunflower-derived products also have an expanding range of food applications, including the use of seed meal as a source of proteins and bioactive compounds in value-added products [34]. Maintaining seed quality is therefore important, but the results of the present study show that relatively stable oil concentration did not compensate for the substantial reduction in seed production observed in 2024, the study year with the lowest mean seed yield.

### 3.3. Cultivar-Related Variation Under Uniform Agronomic Management

Across the three study years, cultivar-related variation was trait-dependent. The repeated-measures analysis detected significant cultivar effects for plant density at harvest, number of seeds per capitulum, seed weight per capitulum, thousand-grain weight (TGW), and seed yield, whereas cultivar effects were not significant for test weight, seed moisture, or oil content. Across years, Averon SU recorded the highest mean seed yield (1513.06 kg ha^−1^), followed by Helesun (1385.22 kg ha^−1^) and Demetra (1297.55 kg ha^−1^). However, the significant cultivar × year interaction for seed yield indicates that cultivar differences should also be interpreted within individual study years rather than solely from the overall cultivar means.

The year-specific cultivar means showed a consistent numerical ranking for seed yield, although the magnitude of the differences among cultivars varied across study years. Averon SU produced 2348.02 kg ha^−1^ in 2023, 915.15 kg ha^−1^ in 2024, and 1275.99 kg ha^−1^ in 2025, representing the highest numerical mean in each study year. Demetra recorded the lowest numerical seed yield in each year, while Helesun occupied an intermediate position. Importantly, the repeated-measures analysis detected a significant cultivar × year interaction for seed yield, indicating that the magnitude of cultivar differences was not constant across the three study years. All three cultivars also showed a pronounced decline in seed yield between 2023 and 2024, followed by a partial recovery in 2025.

The trait-dependent variation observed among cultivars can be considered in the context of advances in modern sunflower breeding. Commercial hybrids are increasingly selected for combinations of yield potential, agronomic performance, seed quality, and adaptation to contrasting production environments [2]. More recent breeding strategies have expanded these objectives through multi-trait selection, including characteristics associated with seed and oil yield and oil quality [4]. Earlier developments in sunflower biotechnology also contributed to broadening the possibilities for genetic improvement of the crop [3]. Together, these developments provide an important background for understanding the performance of modern commercial sunflower material, although the present experiment was not designed to assess breeding progress directly.

Genetic variation nevertheless remains important for specific sunflower traits, even when differences in final productivity are limited. Ref. [35], for example, demonstrated substantial genotypic variation in achene dormancy and investigated its inheritance in hybrid progeny, showing that genetic background can strongly influence specific seed characteristics. Their results also indicate that the expression of seed-related traits may depend on interactions between inherited characteristics and environmental conditions, including temperature during germination. In the present study, significant cultivar-related differences were detected for several productivity-related traits, while pronounced interannual variation was also evident across the three study years.

Genetic differences among sunflower cultivars may also become more apparent when plants are exposed to specific biotic constraints. Ref. [31] reported differential responses among sunflower genotypes under broomrape infestation, while [36] identified differences among hybrids in their response to white rust under Romanian field conditions. Ref. [30] further emphasized the importance of evaluating and developing sunflower hybrids adapted to current environmental conditions in Romania. These findings show that genetic variability remains relevant for sunflower improvement and are consistent with the trait-dependent cultivar responses observed in the present experiment.

The importance of environmental conditions for genotype expression is also supported by recent evaluations of sunflower germplasm. Ref. [5] reported variation in quantitative traits among sunflower genotypes evaluated under field conditions, highlighting the importance of environmental context when assessing genetic performance. In the present study, the same three cultivars were subjected to consistent agronomic management but showed pronounced differences in productivity among the three study years. The partial RDA further detected a significant joint cultivar effect across the eight standardized response traits after conditioning on study year and block (pseudo-F_2,20_ = 11.49, partial R^2^ = 0.535, exact *p* = 0.0278). This multivariate result complements the trait-specific repeated-measures analysis by showing that cultivar identity was associated with the joint response structure across the evaluated traits, rather than implying that all individual traits differed significantly among cultivars.

The long-term no-till management of the experimental field provides an additional context for interpreting cultivar performance. Conservation-oriented systems can improve soil protection and contribute to maintaining soil conditions favorable for crop production under water-limited environments. Under the conditions of Dobrogea, Ref. [20] reported technological and agronomic advantages associated with no-till management in rapeseed cultivation compared with conventional technology. Such findings support the relevance of conservation agriculture for semi-arid production systems. In the present study, however, all cultivars were grown exclusively under no-till management; therefore, the contribution of no-till itself to cultivar stability cannot be separated from the effects of the other experimental conditions.

From an agronomic perspective, cultivar selection remained relevant under the conditions evaluated in this study, as significant cultivar effects were detected for several productivity-related traits, including seed yield. Averon SU showed the highest overall mean seed yield and the highest numerical mean within each of the three study years, followed by Helesun and Demetra. However, the significant cultivar × year interaction for seed yield demonstrates that the magnitude of these cultivar differences varied among study years and should therefore be considered together with the environmental context in which they were expressed. The significant interaction detected for number of seeds per capitulum further indicates that cultivar responses were not uniform across years for all yield components. For seed weight per capitulum, the cultivar × year interaction detected on the original scale was not retained after log transformation, indicating that this interaction was scale-sensitive and should be interpreted cautiously. The results demonstrate both cultivar-related and interannual variation in sunflower performance under the rainfed no-till conditions evaluated during 2023–2025.

### 3.4. Agronomic Implications of Long-Term No-Till Under Semi-Arid Conditions

The present experiment was conducted within a long-term no-till production system in which soil management and agronomic practices were maintained unchanged throughout the three consecutive growing seasons. Although the experimental design did not include a conventional tillage treatment, the results provide information on sunflower performance within an established conservation agriculture system under the semi-arid conditions of Dobrogea. The pronounced differences recorded among the three study years show that substantial interannual variation in crop productivity persisted under long-term no-till management. The system therefore provided a consistent management background for evaluating interannual and cultivar-related variation in sunflower performance across the environmental conditions observed during the three study years.

The potential benefits of no-tillage for soil functioning have been widely documented. Reduced soil disturbance and the maintenance of crop residues at the soil surface can influence soil structure, water infiltration, evaporation, and water storage, which are particularly relevant under limited and irregular rainfall. Ref. [18] reported improvements in soil hydro-physical properties under no-tillage, while [21] demonstrated that tillage systems influence soil enzyme activities and selected chemical properties. Ref. [19], working with sunflower under Romanian conditions, also identified differences in soil physical properties and crop yield among tillage systems. These findings provide a relevant context for interpreting sunflower performance in the long-term no-till system used in the present experiment.

The response of sunflower to soil management has also been reported under different agroecological conditions. Ref. [16] found that tillage practices influenced sunflower emergence, yield, quality, and fuel consumption, while [17] demonstrated that different tillage practices affected sunflower cultivation within a crop rotation system. Ref. [14] further showed that tillage methods can modify soil moisture content and crop yield, emphasizing the importance of soil management where water availability represents a major production constraint. Ref. [37] similarly reported effects of the main soil tillage systems on sunflower productivity and yield structural elements, while [38] evaluated the influence of different soil tillage practices on sunflower yield under Romanian conditions. Together, these studies show that tillage management can modify both the soil environment and the expression of sunflower productivity, although the magnitude and direction of these responses depend on local soil and climatic conditions.

The long-term management history of the experimental field may therefore be relevant when considering the response observed during the three study years. Crop residues were retained on the soil surface and no primary or secondary tillage operations were performed, maintaining the same conservation-oriented management throughout the experiment. Ref. [15] showed that crop sequence and no-till management can influence biological and establishment processes in sunflower production systems. In the present study, plant density at harvest remained relatively high in 2023 and recovered in 2025 after the decline observed in 2024. This pattern cannot be attributed directly to no-tillage because no alternative tillage system was included, but it shows that satisfactory crop establishment was possible within the long-term no-till system despite the contrasting environmental conditions observed across the three study years.

Evidence obtained from other crops under the pedoclimatic conditions of Dobrogea further supports the relevance of conservation-oriented soil management in this region. Ref. [20] reported technological and economic differences between no-till and conventional management in rapeseed cultivation in the Mereni area. Ref. [39] also compared conventional and no-till systems for pea under the arid conditions of Dobrogea, providing additional evidence that tillage choice can influence crop performance under regional water limitations. In maize, ref. [40] demonstrated that soil tillage systems affected productivity, mechanization efficiency, and economic performance under the conditions of south-eastern Romania. Ref. [23] further showed that tillage-related factors interacted with crop management and hybrid response in maize grown on Chernozem soils. Although these studies involve different crops and, in some cases, different water regimes, they collectively emphasize the importance of soil management as part of crop adaptation strategies in south-eastern Romania.

The results of the present study also indicate the limits of relying on soil conservation alone under severe seasonal water deficit. Seed yield reached 2169.06 kg ha^−1^ in 2023 but declined to 848.08 kg ha^−1^ in 2024 despite the same long-term no-till management, fertilization program, plant density target, crop protection measures, and preceding crop. The approximately 61% difference in seed yield between these two study years shows that substantial interannual variation in productivity occurred despite the consistent conservation-oriented management. This observation is consistent with the descriptive correspondence between precipitation and productivity-related traits illustrated by the climate-related ordination; however, because precipitation varied only at the study-year level and was not independently replicated, this relationship should not be interpreted as an independent estimate of the effect of precipitation.

For semi-arid production areas such as Dobrogea, no-till may therefore be most appropriately considered as one component of a broader adaptation strategy rather than as an isolated response to climatic variability. Conservation of soil moisture, maintenance of crop residues, suitable crop rotations, cultivar selection, and management practices adapted to available water resources can operate together to improve the resilience of rainfed production systems. The present experiment shows that sunflower can be successfully cultivated within a long-term no-till system under the pedoclimatic conditions of Dobrogea, while the pronounced interannual variation observed during 2023–2025 highlights the importance of considering seasonal environmental conditions when evaluating crop performance.

### 3.5. Practical Implications for Sunflower Production in Semi-Arid Environments

The findings of the present study have important practical implications for sunflower cultivation under the increasingly variable climatic conditions characteristic of south-eastern Europe. The pronounced interannual variation observed during the three study years, together with the trait-dependent cultivar responses and significant cultivar × year interactions detected for some productivity-related traits, indicates that improving sunflower productivity in semi-arid environments should consider both cultivar selection and adaptation to variable environmental conditions. This is particularly relevant for South Dobrogea, where oilseed crops have long represented an important component of agricultural production and their cropping structure has changed in response to regional production conditions [41].

The results further emphasize that successful sunflower production should be approached as an integrated production system rather than through the optimization of a single agronomic factor. Cultivar selection remains important, but its benefits should be combined with appropriate soil management, crop rotation and balanced nutrient supply. Diversified crop rotations can improve agronomic and economic performance and contribute to the resilience of agricultural systems under variable environmental conditions [24]. In the present study, sunflower was cultivated after chickpea, providing a legume–oilseed sequence within the long-term no-till system. The importance of crop diversification under water-limited conditions has also been recognized in southern Romania, where [42] evaluated safflower as an alternative crop to sunflower for arid environments.

Nutrient management represents another component that can influence the capacity of sunflower to maintain productivity under limiting environmental conditions. Ref. [43] demonstrated that soil-applied sulphur and zinc can modify sunflower hybrid growth, achene yield and oil yield, illustrating the interaction between plant nutrition and hybrid performance. Similarly, ref. [44] reported differences in sunflower yield and economic performance in response to nutrient management. These findings are relevant to the present study because they indicate that, although seasonal climatic variability cannot be controlled directly, management of crop nutrition represents an agronomic component that can be adjusted according to production conditions. The fixed fertilization program used throughout the present experiment provided comparable nutritional management across cultivars and study years, while future studies could investigate whether adaptive nutrient management can improve sunflower performance during contrasting growing seasons.

Climate adaptation strategies should also consider biotic constraints, particularly because environmental variability may occur simultaneously with changes in disease and parasite pressure. Ref. [45] documented the evolution of pathogens and broomrape virulence in sunflower crops in Dobrogea, demonstrating that regional sunflower production is exposed to biological constraints in addition to drought and high temperatures. Consequently, cultivar selection should not be based exclusively on yield potential under water-limited conditions but should also consider resistance or tolerance to the principal biotic stresses occurring in the production area. This reinforces the need for an integrated strategy in which genetic characteristics, climatic adaptation, crop protection and agronomic management are considered together.

The increasing availability of digital technologies provides additional opportunities for implementing such adaptive management. Ref. [46] demonstrated the applicability of Sentinel-1 time-series data for monitoring sunflower phenology, providing a means of following crop development throughout the growing season. More recent approaches based on deep learning have extended these possibilities to sunflower mapping and stand counting, although methodological trade-offs remain between different data-access and analytical approaches [47]. Such tools could be particularly useful in years characterized by irregular crop establishment or rapid changes in environmental conditions, allowing spatial and temporal variability to be identified before final yield is determined.

At the regional scale, these monitoring approaches could be incorporated into broader decision-support systems. Ref. [48] proposed a theoretical model for implementing digital twins in farms from the Dobrogea Region, providing a framework for integrating field information into digital farm management. The combination of meteorological monitoring, crop observations, remote sensing and field-level agronomic data could allow management decisions to respond more rapidly to seasonal variability. Although digital technologies were not evaluated in the present experiment, the pronounced interannual differences identified here highlight the potential value of tools capable of detecting climatic and crop responses during the growing season rather than only after harvest.

Future adaptation strategies should also consider environmental sustainability beyond crop productivity. Evidence from other rainfed cropping systems in Dobrogea also highlights the importance of crop and management adaptation for maintaining agricultural resilience under variable climatic conditions [49]. Sunflower fields form part of wider agroecosystems in which crop protection practices can affect non-target organisms and agricultural products beyond the harvested seed. Ref. [50], for example, compared pesticide residues in hive products associated with rapeseed and sunflower crops under different agricultural practices in Romania, illustrating the wider environmental implications of crop protection management. Thus, improving sunflower resilience should not lead to an indiscriminate intensification of chemical inputs. Conservation-oriented soil management, appropriate crop protection and efficient nutrient use need to be considered together when developing production systems capable of maintaining productivity while limiting environmental impacts.

The present results therefore have implications beyond the comparison of three sunflower cultivars. They show that, under a common long-term no-till management system, interannual differences in precipitation and temperature were accompanied by substantial changes in crop establishment, yield formation, and final productivity, while cultivar-related variation was trait-dependent and, for some productivity-related traits, varied among study years. For semi-arid environments such as Dobrogea, adaptation strategies may therefore combine cultivar selection with soil water conservation, diversified crop rotations, balanced nutrient management, appropriate crop protection, and increasingly precise monitoring of crop and climatic conditions. Digital tools may further improve the timing and spatial targeting of these interventions, but they should complement rather than replace sound agronomic management.

From a practical perspective, the results support the relevance of management strategies capable of responding to interannual environmental variability at several levels. Long-term conservation-oriented soil management can provide the production framework, while cultivar selection, crop rotation, nutrient management and crop protection can be adjusted to specific field conditions. Remote sensing and digital decision-support systems may further assist this process by providing information on crop development and spatial variability during the growing season. Such an integrated approach is consistent with the pronounced interannual and cultivar-related variation observed in the present study and provides a relevant framework for managing sunflower production under increasingly variable semi-arid conditions without relying on any single technological intervention.

## 4. Materials and Methods

### 4.1. Study Site

The field experiment was conducted over three consecutive growing seasons (2023–2025) in Mereni, Constanța County, in the Dobrogea Region of south-eastern Romania (43°57′ N, 28°02′ E; approximately 180 m a.s.l.). The experimental area is representative of the semi-arid agroecological conditions of Dobrogea, where sunflower (*Helianthus annuus* L.) is widely cultivated under predominantly rainfed conditions. The regional climate is characterized by relatively low and irregular precipitation, high evaporative demand during the growing season, and recurrent drought episodes, particularly during late spring and summer.

The experiment was maintained within the same commercial agricultural field throughout the three-year study period to minimize variation associated with soil type, field history, and topographic position. The field had been managed under a long-term no-till production system prior to the establishment of the experiment and remained under no-till management throughout the study. Chickpea (*Cicer arietinum* L.) was used as the preceding crop before sunflower in each experimental year. Agronomic management, including sowing practices, fertilization, crop protection, and harvesting procedures, was maintained consistently across the three growing seasons. This consistent management framework provided a common agronomic background for evaluating interannual variation in sunflower performance and cultivar responses across the environmental conditions observed during the three study years.

Meteorological conditions during each growing season were characterized using data recorded by an automatic weather station (Davis Instruments Corp., Hayward, CA, USA) located in close proximity to the experimental field. Monthly precipitation and mean air temperature were recorded from March to August in each experimental year (2023–2025). A detailed description of meteorological data acquisition, long-term climatic reference values, and the procedures used for climatic comparisons is provided in Section 2.5.

### 4.2. Soil Characteristics

The experiment was established on a Chernozem developed on loess deposits, a soil type widely distributed in the Dobrogea Region and representative of the agricultural soils of south-eastern Romania. The experimental field had a relatively uniform topography and good natural drainage. The groundwater table remained below the active rooting zone during the study period and did not contribute to crop water supply during the growing season.

Before the experiment was established, composite soil samples were collected from the 0–30 cm soil layer to determine the main physical and chemical properties of the soil. The analyses included soil pH, organic matter content, total nitrogen, available phosphorus, available potassium, calcium carbonate content, and particle-size distribution. Soil particle-size distribution was determined using the Kachinsky method [51]. Soil pH was measured potentiometrically in aqueous suspension, organic matter content was determined using the Walkley–Black method [52], and total nitrogen was determined by the Kjeldahl method [53]. Available phosphorus and potassium were extracted using the Egner–Riehm–Domingo (AL) method [54], while calcium carbonate content was determined using the Scheibler gas-volumetric method [55].

The soil had a pH of 6.69, an organic matter content of 3.42%, and a total nitrogen content of 0.201%. Available phosphorus and potassium were 5.82 and 186 mg kg^−1^, respectively. Calcium carbonate was not detected in the analyzed 0–30 cm soil layer. The soil had a clay loam texture, with 41.4% sand, 30.2% silt, and 28.4% clay. These soil characteristics were representative of the field conditions in which the three sunflower cultivars were evaluated during the 2023–2025 growing seasons.

### 4.3. Plant Material

Three commercial sunflower (*Helianthus annuus* L.) hybrids, Averon SU, Demetra, and Helesun, were evaluated in this study. The hybrids were selected because they were recommended for cultivation under semi-arid conditions, are suitable for the pedoclimatic conditions of the Dobrogea Region, and are used in rainfed sunflower production in south-eastern Romania. Their inclusion allowed cultivar-related differences in productivity and seed quality to be evaluated under the same rainfed no-till management and climatic conditions. The hybrids were not selected to represent contrasting water, soil, or light requirements, and these characteristics were not experimentally evaluated in the present study. All three hybrids are tolerant to sulfonylurea (SU) herbicides, allowing post-emergence weed control with tribenuron-methyl. The same hybrids were evaluated during each of the three growing seasons (2023–2025). Sowing date, target plant density, fertilization, crop protection measures, and harvesting procedures were kept the same for all hybrids and experimental years.

The use of the same plant material throughout the experimental period allowed the performance of each hybrid to be followed under the contrasting climatic conditions recorded from 2023 to 2025. This approach provided a common basis for comparing cultivar responses across the three study years and for examining interannual and cultivar-related variation in the measured agronomic and seed quality traits. The hybrids were evaluated under rainfed field conditions without supplemental irrigation, so their performance reflected the water and temperature conditions occurring naturally during each growing season.

### 4.4. Experimental Design and Crop Management

**Figure 8:**
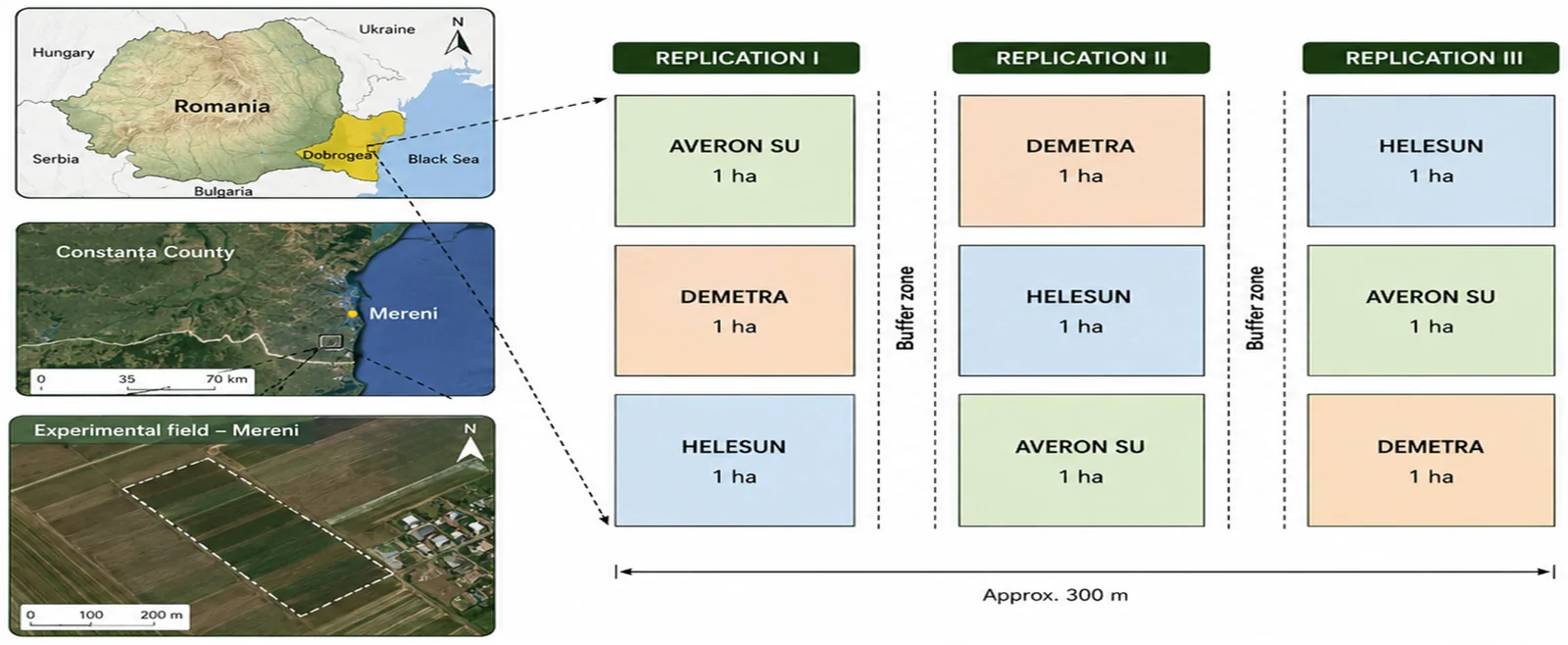
Geographic location of the experimental field and layout of the field experiment established under rainfed no-till conditions in Mereni, Constanța County, Romania. Three sunflower hybrids (Averon SU, Demetra and Helesun) were evaluated in a randomized complete block design comprising three blocks, with each hybrid represented once within each block and each experimental plot covering 1 ha.

The field experiment was conducted at Mereni, Constanța County, in the southern part of the Dobrogea Region, south-eastern Romania, at an approximate elevation of 74 m above sea level. The experimental site is located within the semi-arid agricultural area of southern Dobrogea. The experiment was conducted over three consecutive growing seasons (2023–2025) using three commercial sunflower (*Helianthus annuus* L.) hybrids, namely Averon SU, Demetra, and Helesun, cultivated under the same agronomic conditions. A randomized complete block design (RCBD) was used, comprising three blocks, with each of the three hybrids represented once within each block. Each experimental plot covered 1 ha, resulting in a total experimental area of 9 ha per study year (3 hybrids × 3 blocks). The same experimental structure was maintained during each of the three study years, resulting in 27 plot-year observations across the complete 2023–2025 dataset. The large plot size was selected to reflect commercial farming conditions, minimize border effects, and allow all field operations to be performed using commercial farm machinery. Figure 8 shows the geographical location of the experimental field together with the layout of the experimental design.

All experimental plots were established within the same agricultural field. The experimental design and crop management practices were maintained throughout the three-year study period to minimize variation associated with field history and agronomic management. The experiment was conducted entirely under rainfed conditions, without supplemental irrigation. Sowing date, target plant density, fertilization, crop protection measures, and harvesting management were kept the same during the three experimental seasons. This approach allowed the performance of the three hybrids to be evaluated under the climatic conditions occurring naturally in each growing season. The experimental field had been continuously managed under no-till since 2019, prior to the establishment of the present experiment in 2023. Chickpea was the crop immediately preceding sunflower, and its residues were retained on the soil surface after harvest.

Sunflower was cultivated under a continuous no-till production system in which no primary or secondary tillage operations were performed before sowing. Seeds were directly drilled into the chickpea stubble remaining after harvest using a precision planter (CNS 2000, Cansa Tarım Makinaları, Ceyhan, Adana, Türkiye) adapted for direct seeding under conservation agriculture. Crop residues were retained on the soil surface throughout the experimental period, maintaining the characteristics of the no-till production system and reducing mechanical soil disturbance. Sowing was performed on 25 March in each experimental year using a row-crop precision planter adjusted to establish a target population of approximately 56,000 plants ha^−1^, a density selected for rainfed sunflower production under the semi-arid conditions of the Dobrogea Region. At sowing, a compound mineral fertilizer containing 15% N, 15% P_2_O_5_, and 15% K_2_O was applied at a rate of 280 kg ha^−1^.

Weed control was carried out during the early vegetative stages using tribenuron-methyl applied together with an adjuvant for the control of broadleaf weeds. This herbicide program was used because all three sunflower hybrids were tolerant to sulfonylurea herbicides. Crop protection included two phytosanitary treatments performed during each growing season. The first application consisted of a fungicide containing azoxystrobin and difenoconazole, combined with an insecticide based on lambda-cyhalothrin, for the control of fungal diseases and early-season insect pests occurring in the sunflower crop. A second treatment was applied later during crop development using the systemic insecticide acetamiprid together with a foliar fertilizer containing macro- and micronutrients to support plant nutritional status during reproductive development. The same weed and crop protection program was applied to all hybrids and experimental plots during the three growing seasons.

Harvesting was performed mechanically between 26 and 29 August in each experimental year using a combine harvester equipped with a sunflower header. Harvest operations were initiated when plants reached physiological maturity and seed moisture declined below 13%. The same maturity and moisture criteria were used to determine the appropriate harvesting period in each year. Each experimental plot was harvested separately, and seed yield was determined for each plot and expressed on a unit-area basis. Representative seed samples were collected from each experimental plot after harvest for the subsequent laboratory determination of yield components and seed quality parameters. The 1-ha plot size allowed sowing, crop protection, and harvesting to be performed using commercial farm machinery under practical production conditions while maintaining the randomized complete block structure within each study year.

### 4.5. Meteorological Data

Meteorological data were collected throughout the experimental period using an automatic weather station (Davis Instruments Corp., Hayward, CA, USA) installed in close proximity to the experimental field. Precipitation (mm) and air temperature (°C) were recorded continuously, and monthly precipitation totals and mean monthly air temperatures were calculated for the sunflower growing season, from March to August, in each experimental year (2023–2025). The March–August period was selected to cover the main field cycle of the crop, from sowing and early establishment to reproductive development and harvest.

To characterize the climatic conditions of the three experimental seasons, monthly precipitation and mean air temperature values were compared with the corresponding long-term climatic averages representative of the Dobrogea Region. The multiannual climatic reference covered a 50-year period and was used as a common baseline for evaluating the climatic conditions recorded in 2023, 2024, and 2025. Monthly deviations from the long-term precipitation and temperature values were examined to identify differences in rainfall availability and thermal conditions during crop development.

The meteorological data were used to describe the environmental conditions experienced by the sunflower crop during each growing season and to examine their relationship with agronomic performance and seed quality. Particular attention was given to differences in precipitation amount and distribution among years, together with changes in mean air temperature during the March–August period. Precipitation and temperature had only three unique year-level values and were common to all experimental plots within each study year. Consequently, these variables were not treated as independently replicated explanatory variables in the inferential multivariate analysis but were used to characterize the environmental conditions represented by the three study years.

### 4.6. Agronomic and Seed Quality Measurements

Agronomic measurements were performed at physiological maturity using representative samples collected from each experimental plot. All determinations were carried out independently for each experimental plot, and the resulting data were subsequently used for statistical analysis. Plant density at harvest was determined by counting all surviving plants along representative 10 m row sections located in the central part of each experimental plot to minimize border effects. Plant density was expressed as the number of plants per 10 m of row (plants 10 m^−1^ row).

Seed yield was determined separately for each experimental plot after mechanical harvesting and expressed as kg ha^−1^. Representative sunflower capitula were randomly collected from each replication for the determination of yield components. The number of fully developed seeds per capitulum was determined by manual counting after threshing, whereas seed weight per capitulum (g) was measured using an analytical balance.

Thousand Grain Weight (TGW) was determined according to the International Seed Testing Association (ISTA) methodology using randomly selected seeds from each experimental sample. Test weight was expressed as kg hL^−1^ and determined using an ML-Hecto 100 grain tester (Tripette & Renaud, Asnières-sur-Seine, France). Seed moisture (%) was measured immediately after harvest and before subsequent laboratory analyses.

Seed oil content (%) was determined by Soxhlet extraction using petroleum ether as the extraction solvent and was expressed on a dry matter basis. All agronomic, yield component, and seed quality measurements were obtained separately for each experimental plot within each block to preserve the structure of the experimental design for subsequent statistical analyses.

### 4.7. Statistical Analyses

All statistical analyses were performed using R software version 4.5.1 (R Foundation for Statistical Computing, Vienna, Austria). Before statistical analysis, the datasets were examined for normality and homogeneity of variances to verify the assumptions required for parametric statistical procedures. Monthly precipitation and mean air temperature recorded from March to August during each growing season were compared with the corresponding long-term climatic reference values using paired-sample *t*-tests. The monthly values represented the paired observations for these comparisons. Statistical significance was accepted at *p* < 0.05.

Variation in agronomic performance, seed yield, yield components, and seed quality traits was evaluated using a repeated-measures analysis of variance, with study year (2023, 2024, and 2025) treated as the within-unit repeated factor and cultivar (Averon SU, Demetra, and Helesun) as the between-unit factor. The experimental design comprised three blocks, with each cultivar represented once within each block, and cultivar effects were tested while accounting for block. The model included the main effects of study year and cultivar and the cultivar × year interaction. For effects involving the repeated year factor, Greenhouse–Geisser-adjusted degrees of freedom and *p*-values were used to account for departures from sphericity. Cultivar effects were tested with 2 and 4 numerator and denominator degrees of freedom, respectively. When significant effects were detected, Tukey-adjusted pairwise comparisons were used to separate means at *p* < 0.05. Results are presented as mean ± standard deviation (SD), and different lowercase letters indicate statistically significant differences among means according to the Tukey-adjusted comparisons. Because the six-monthly observations within each year were temporally ordered and may not be fully independent, these paired comparisons were used as descriptive comparisons with the long-term monthly reference rather than as evidence for classifying individual study years as drought treatments. Seasonal precipitation totals and their distribution during the growing period were therefore considered directly when interpreting sunflower performance.

Relationships among agronomic traits, yield components, and seed quality parameters were examined using Pearson’s correlation analysis.

To describe cultivar performance relative to the corresponding yearly conditions, cultivar means were also expressed as percentage deviations from the corresponding yearly mean for each measured trait. Deviations were calculated separately for each cultivar, year, and trait and were subsequently averaged across the three experimental years. Positive values indicated cultivar performance above the corresponding yearly mean, whereas negative values indicated values below the yearly mean.

For the inferential multivariate analysis of cultivar effects, a partial redundancy analysis (partial RDA) was performed using a response matrix containing 27 plot-year observations and eight standardized agronomic and seed quality traits. Cultivar identity was included as the constrained explanatory variable, while study year and block were included as conditioning variables to account for their associated variation before testing the cultivar component. Cultivar was treated as a three-level categorical factor and was represented by two indicator degrees of freedom, with Averon SU used as the reference level for coefficient coding.

The significance of the cultivar component was assessed using a design-based permutation procedure that preserved the experimental structure. Permutations were restricted to whole cultivar plots within blocks, and the same cultivar reassignment was maintained across all three study years. Enumeration yielded 216 permissible allocations, including the observed allocation, allowing an exact design-based *p*-value to be calculated. The partial RDA therefore tested the joint multivariate cultivar signal across the eight standardized response traits after conditioning on study year and block. Precipitation and mean air temperature were not included as inferential explanatory variables in this model because each had only three unique year-level values, which were repeated across plots within a given year and were therefore inseparable from study year. Consequently, the original inferential tests from the climate-constrained RDA, including the unrestricted 999-permutation tests, were not retained. Climatic variables were used only for descriptive characterization and visualization of the environmental differences among the three study years and were not interpreted as providing independent estimates of precipitation or temperature effects. For descriptive visualization, the original climate-related RDA ordination was retained to illustrate the multivariate correspondence among the environmental conditions of the three study years, cultivar identity, and the standardized agronomic and seed quality traits. In this descriptive ordination, the year-level precipitation and temperature values were repeated across plots only to position the observations within the common ordination space. Because these climatic variables had only three unique values and were confounded with study year, no permutation-based significance tests or independent climatic effect estimates were derived from this ordination. Accordingly, the lengths and directions of the climatic vectors were interpreted only as descriptive associations with the multivariate trait structure observed during 2023–2025.

Graphical representations were generated to facilitate interpretation of the experimental data and comparison among growing seasons and cultivars. Parallel coordinate plots were used to visualize variation in the measured traits among years and cultivars, while raincloud plots were used to display the distribution of precipitation and temperature. Heatmaps were used to compare relative mean values of agronomic and seed quality parameters. Cultivar deviation plots were used to visualize cultivar-specific responses relative to the corresponding yearly means, and radar charts were used to compare the normalized response of climatic, agronomic, yield, and seed quality variables across the three growing seasons. The descriptive RDA biplot was used to visualize the multivariate correspondence among the environmental conditions of the three study years, cultivars, and sunflower performance traits.

## 5. Conclusions

This three-year field study showed substantial interannual and cultivar-related variation in sunflower productivity under rainfed long-term no-till conditions in the semi-arid Dobrogea Region of Romania. Significant interannual variation was detected across all eight evaluated traits, while significant cultivar effects were identified for plant density at harvest, number of seeds per capitulum, seed weight per capitulum, thousand-grain weight, and seed yield. Significant cultivar × year interactions were also detected for number of seeds per capitulum and seed yield, indicating that cultivar responses for these traits were not uniform across the three study years. For seed weight per capitulum, the cultivar × year interaction detected on the original scale was not retained after log transformation and should therefore be interpreted cautiously.

The three study years showed contrasting patterns of sunflower performance. The more favorable precipitation conditions recorded in 2023 were associated with higher plant density, seed number per capitulum, seed weight per capitulum, thousand grain weight, test weight, and seed yield. Conversely, the markedly lower precipitation recorded in 2024 coincided with substantial reductions in all major yield components and a seed yield approximately 61% lower than that obtained in 2023. The partial recovery observed in 2025 coincided with environmental conditions that differed from those recorded in 2024, illustrating the marked interannual variation in sunflower performance across the three-year dataset.

The descriptive climate-related ordination showed a close correspondence between precipitation and several productivity-related traits within the three-year dataset. However, because precipitation and temperature had only three unique year-level values and were common to all experimental plots within each year, these relationships should be interpreted as descriptive associations rather than as independently replicated climatic effects. In contrast to the pronounced interannual variation observed for yield and its components, oil content remained comparatively stable across the three study years, indicating that seed oil concentration varied less across the observed study years than productivity-related traits.

The results also show that substantial yield reductions occurred during the study year characterized by the lowest March–August precipitation total, despite the established long-term no-till production system. Because no alternative tillage treatment was included, the present study does not allow the specific contribution of no-till to drought mitigation to be quantified. Nevertheless, the findings show that sunflower can be cultivated under long-term no-till management in the semi-arid conditions of Dobrogea, while substantial variation in productivity was observed across the contrasting environmental conditions represented by the three study years.

From a practical perspective, the pronounced interannual variation observed during the study, together with the significant cultivar effects detected for several productivity-related traits, highlights the importance of considering both cultivar selection and seasonal environmental variability when evaluating sunflower production under semi-arid conditions. The significant cultivar × year interactions observed for seed number and seed yield further indicate that cultivar performance should be interpreted within the environmental conditions of individual study years rather than solely on the basis of overall cultivar means.

This study provides field-based evidence that, under the specific environmental conditions observed during 2023–2025 in the Dobrogea Region of south-eastern Romania, sunflower performance showed pronounced interannual variation together with trait-dependent cultivar responses. The partial RDA further detected a significant joint cultivar effect across the eight standardized response traits after conditioning on study year and block (pseudo-F_2,20_ = 11.49, partial R^2^ = 0.535, exact *p* = 0.0278). Because the climatic conditions of each year were common to all experimental plots and were not independently replicated, the observed relationships with precipitation and temperature should not be interpreted as independent climatic effects or as a causal ranking of climatic and cultivar influences. These findings contribute to understanding sunflower performance across contrasting study years and support the importance of considering both cultivar-related and interannual environmental variation when developing production strategies for water-limited agricultural environments.

## Figures and Tables

**Table 1:** Paired-sample *t*-test results comparing precipitation and mean air temperature during the 2023–2025 growing seasons with the corresponding long-term climatic reference values.

Indicator	Yearly Values vs. Normal Values	Difference	t	*p*
Precipitations	2023	5.25	0.34	0.74
	2024	19.07	3.01	0.03
	2025	17.23	2.25	0.07
Temperature	2023	−3.07	−5.90	0.002
	2024	−3.07	−5.90	0.002
	2025	−3.25	−3.54	0.017

Differences represent the mean paired differences calculated as the long-term reference values minus the corresponding monthly values recorded from March to August. t, paired-sample *t*-test statistic; *p*, probability value. Values with *p* < 0.05 indicate statistically significant differences.

**Table 2:** Mean values (± SD) of agronomic and seed quality traits according to sunflower cultivar and study year during the 2023–2025 experimental period.

Parameter	Averon SU	Demetra	Helesun	2023	2024	2025
Oil content (%)	45.53 ± 0.93 a	46.09 ± 0.95 a	46.37 ± 1.18 a	46.88 ± 0.82 a	45.19 ± 0.79 c	45.92 ± 0.80 b
Plant density at harvest (plants 10 m^−1^ row)	48.11 ± 5.04 ab	45.89 ± 4.54 b	48.89 ± 4.96 a	50.56 ± 2.19 a	41.33 ± 1.22 b	51.00 ± 1.66 a
Seed weight per capitulum (g)	35.34 ± 4.35 a	31.49 ± 3.85 b	32.18 ± 3.81 b	37.88 ± 2.13 a	28.77 ± 1.63 c	32.36 ± 1.77 b
Thousand-grain weight (g)	41.73 ± 3.73 a	40.41 ± 3.63 b	41.79 ± 3.78 a	45.67 ± 0.78 a	37.14 ± 0.70 c	41.12 ± 0.83 b
Test weight (kg hL^−1^)	40.44 ± 3.34 a	39.48 ± 3.20 a	40.14 ± 3.20 a	43.64 ± 0.40 a	36.28 ± 0.64 c	40.13 ± 1.05 b
Number of seeds per capitulum	965.00 ± 194.38 a	798.67 ± 160.56 b	979.67 ± 195.10 a	1138.00 ± 112.48 a	721.89 ± 73.20 c	883.44 ± 85.60 b
Seed yield (kg ha^−1^)	1513.06 ± 646.24 a	1297.55 ± 565.81 c	1385.22 ± 575.00 b	2169.06 ± 143.26 a	848.08 ± 59.55 c	1178.68 ± 87.01 b
Seed moisture (%)	8.81 ± 0.47 a	8.92 ± 0.58 a	9.00 ± 0.53 a	9.36 ± 0.34 a	8.44 ± 0.40 b	8.94 ± 0.32 ab

Values are presented as mean ± standard deviation (SD). Within each factor (cultivar and study year), means followed by different lowercase letters indicate significant differences according to Tukey-adjusted pairwise comparisons at *p* < 0.05. Overall effects of year, cultivar, and cultivar × year are presented in Table 3.

**Table 3:** Repeated-measures analysis of year, cultivar, and cultivar × year effects on sunflower agronomic and seed quality traits during the 2023–2025 study period.

Parameter	Year F (df_1_, df_2_)	*p*	Cultivar F	*p*	Cultivar × Year F (df_1_, df_2_)	*p*
Plant density at harvest (plants 10 m^−1^ row)	185.56 (1.06, 6.34)	**<0.001**	11.33	**0.022**	0.22 (2.11, 6.34)	0.8205
Number of seeds per capitulum	3217.18 (1.09, 6.53)	**<0.001**	38.44	**0.002**	11.17 (2.18, 6.53)	**0.00729**
Seed weight per capitulum (g)	4382.34 (1.23, 7.40)	**<0.001**	44.54	**0.001**	6.98 (2.47, 7.40)	**0.0170**
Thousand-grain weight (g)	8500.73 (1.48, 8.87)	**<0.001**	36.20	**0.002**	2.62 (2.96, 8.87)	0.1162
Test weight (kg hL^−1^)	392.17 (1.38, 8.29)	**<0.001**	5.00	0.081	0.39 (2.76, 8.29)	0.7463
Seed yield (kg ha^−1^)	31,082.38 (1.77, 10.60)	**<0.001**	71.51	**0.0007**	56.90 (3.53, 10.60)	**<0.001**
Seed moisture (%)	10.52 (1.76, 10.55)	**0.00373**	3.80	0.118	0.59 (3.52, 10.55)	0.6609
Oil content (%)	4915.20 (1.08, 6.48)	**<0.001**	6.26	0.058	0.50 (2.16, 6.48)	0.6421

Year was treated as the within-unit repeated factor and cultivar as the between-unit factor. Cultivar effects were tested while accounting for block, with 2 and 4 numerator and denominator degrees of freedom, respectively. Greenhouse–Geisser-adjusted degrees of freedom and *p*-values are reported for year and cultivar × year effects. Significant effects (*p* < 0.05) are shown in bold. For seed weight per capitulum, the cultivar × year interaction was significant on the original scale (*p* = 0.0170) but was not retained after log transformation (*p* = 0.619).

**Table 4:** Cultivar-specific mean seed yield within each study year during the 2023–2025 experimental period.

Study Year	Averon SU (kg ha^−1^)	Helesun (kg ha^−1^)	Demetra (kg ha^−1^)
2023	2348.02	2127.23	2031.94
2024	915.15	848.01	781.09
2025	1275.99	1180.42	1079.63

Values represent mean seed yield (kg ha^−1^) for each cultivar within each study year. The overall repeated-measures analysis detected a significant cultivar × year interaction for seed yield (Greenhouse–Geisser-adjusted F_3.53,10.60_ = 56.90, *p* < 0.001; Table 3). Because adjusted pairwise comparison statistics for the cultivar simple effects within individual years were not available, significance letters are not assigned to the year-specific cultivar means in this table.

## Data Availability

The original contributions presented in this study are included in the article. Further inquiries can be directed to the corresponding authors.

## Abbreviations

The following abbreviations are used in this manuscript:

ANOVA	Analysis of Variance
HSD	Honestly Significant Difference
RDA	Redundancy Analysis
TGW	Thousand Grain Weight
SD	Standard Deviation
SU	Sulfonylurea
R^2^	Coefficient of Determination
